# Screening of Health-Associated Oral Bacteria for Anticancer Properties *in vitro*

**DOI:** 10.3389/fcimb.2020.575656

**Published:** 2020-10-06

**Authors:** Divyashri Baraniya, Vinay Jain, Ronald Lucarelli, Vincent Tam, Lisa Vanderveer, Sumant Puri, Maobin Yang, Nezar Noor Al-hebshi

**Affiliations:** ^1^Oral Microbiome Research Laboratory, Department of Oral Health Sciences, Maurice H. Kornberg School of Dentistry, Temple University, Philadelphia, PA, United States; ^2^Department of Microbiology and Immunology, Lewis Katz School of Medicine, Temple University, Philadelphia, PA, United States; ^3^Fox Chase Cancer Center, Temple University Health System, Philadelphia, PA, United States; ^4^Regenerative Research Laboratory, Department of Endodontology, Maurice H. Kornberg School of Dentistry, Temple University, Philadelphia, PA, United States

**Keywords:** bacteria, carcinoma, cell line, cell proliferation, mouth, coculture techniques, gene expression

## Abstract

While extensive literature exists about the role of oral bacterial pathogens like *Porphyromonas gingivalis* and *Fusobacterium nucleatum* in oral squamous cell carcinoma (OSCC), the role of health-associated species has been largely unexplored. In this study, we assessed the effect of *Streptococcus mitis, Rothia mucilaginosa, Neisseria flavescens, Haemophilus parainfluenzae, Lautropia mirabilis*, and *Veillonella parvula* on proliferation and expression of marker genes (IL-6, TNF-α, MMP3, CD36, CCD1, and NANOG) in OSCC cell lines CAL27, SCC25, and SCC4. *Porphyromonas gingivalis* was included as a pathogenic control. Both bacterial lysates (3 concentrations) and live cells (3 MOIs) were tested. *S. mitis, H. parainfluenzae*, and *N. flavescens* resulted in substantial, dose-dependent reduction of proliferation, which was found to be mediated by H_2_O_2_ for the former and intracellular infection in the latter two species. However, only *H. parainfluenzae* showed differential antiproliferative effect against the cancer cell lines vs. the normal control (TIGKs). In the gene expression assays, the health-associated species mostly downregulated CD36, a gene that plays an important role in tumor growth and metastasis, while *P. gingivalis* upregulated it. IL6 and TNF expression, on the other hand, was upregulated by almost all species, particularly the Gram-negatives including *P. gingivalis*. The effect on other genes was less evident and varied significantly by cell line. This exploratory study is the first insight into how health-associated bacteria may interact with OSCC. Further studies to explore whether the observed effects may have implications for the prevention or treatment of oral cancer are warranted.

## Introduction

Oral cancer, predominantly oral squamous cell carcinoma (OSCC), continues to be a major global health burden, with poor prognosis and a 5-year survival rate of <50%. It accounts for 354,864 new cases and 177,384 deaths globally each year (Bray et al., 2018). There is a male predilection, and the tongue is the most commonly affected subsite (Siegel et al., 2017). Use of smoked and smokeless forms of tobacco, alcohol consumption, and betelnut chewing are the major risk factors (Gupta et al., 2016), whereas 2–6% of the cases are attributed to human papilloma virus (HPV) (Lingen et al., 2013). However, about 15% of all OSCC cases are not associated with any of the known risk factors (Chocolatewala et al., 2010).

Recently, there has been increasing interest in the potential role of the microbiome in oral cancer. In a plethora of clinical studies, analyses of samples from OSCC patients have revealed significant differences in microbiome composition and function between tumor and control samples, with certain taxa quite consistently showing association with OSCC across studies (Perera et al., 2016; Al-Hebshi et al., 2019). Of these, *Fusobacterium nucleatum* and *Porphyromonas gingivalis* have received the most attention, with large number of studies that characterized their carcinogenic properties *in vitro* and in *in vivo* models (Fitzsimonds et al., 2020). However, in focusing on exploring the role of species that are enriched in the tumors, i.e., the pathobionts, researchers seem to have largely ignored the health-associated species that in fact may be playing a protective role against oral cancer. Indeed, Ohshima et al. (2019) have very recently found *Streptococcus gordonii*, a health-associated species, to antagonize the pro-cancer phenotypes induced by *P. gingivalis*, highlighting the need to explore the other side of the relationship between the microbiome and oral cancer.

A number of bacterial taxa have consistently been found to be more abundant in samples from healthy subjects compared to those from cancer/precancer patients. These include *Streptococcus spp*. such as *Streptococcus mitis* (Pushalkar et al., 2012; Schmidt et al., 2014; Al-Hebshi et al., 2017; Amer et al., 2017), *Haemophilus spp*., mainly *Hemophilus parainfluenzae* (Al-Hebshi et al., 2017; Guerrero-Preston et al., 2017; Perera et al., 2017), *Neisseria spp*. (Al-Hebshi et al., 2017; Amer et al., 2017; Guerrero-Preston et al., 2017; Hayes et al., 2018), *Lautropia spp*., mostly *L. mirabilis* (Al-Hebshi et al., 2017; Amer et al., 2017; Perera et al., 2017; Zhao et al., 2017) *Veillonela spp*. (Al-Hebshi et al., 2017; Zhao et al., 2017) and *Rothia spp*. (Schmidt et al., 2014; Al-Hebshi et al., 2017; Zhao et al., 2017), although for the latter there are studies that show an association with cancer (Amer et al., 2017; Mukherjee et al., 2017). One study even found the relative abundances of *S. mitis, H. parainfluenzae*, and *Veillonella parvula* to inversely correlate with oral cancer staging (Yang et al., 2018). None of these presumably health-associated taxa has been screened for its effect on epithelium or OSCC cell lines yet.

The objective of this study was to assess the *in vitro* effects of a panel of oral health-associated bacterial species, identified from previous microbiome studies, on oral squamous cell carcinoma (OSCC) cell lines. Namely *S. mitis, Rothia mucilaginosa, Veillonella parvula, Neisseria flavescens, H. parainfluenzae*, and *L. mirabilis*, were individually screened for their effect on proliferation and gene expression in OSCC cell lines CAL27, SCC25, and SCC4, in comparison with. *P. gingivalis* as a carcinogenic species. The gene panel included markers of inflammation (IL-6 and TNF-α), invasion and metastasis (MMP3 and CD36), proliferation (CCND1), and stemness (NANOG).

## Materials and Methods

### Bacterial Strains and Culture Conditions

*Porphyromonas gingivalis* ATCC 33277, *Veillonella parvula* ATCC 17745, *Streptococcus mitis* ATCC 49456, *Neisseria flavescens* ATCC 13120, *Hemophilus parainfluenzae* NCTC 10665*, Rothia mucilaginosa* ATCC 49041, and *Lautropia mirabilis* ATCC 51599 were obtained from the American Tissue Culture Collection (ATCC) or Public Health England. *V. parvula* was cultivated in Brain Heart Infusion (BHI) supplemented with 1.5 of 60% sodium lactate. All other bacteria were cultivated in BHI supplemented with 0.5% hemin, 0.1% Vitamin K and 1% Isovitalex. *P. gingivalis* and *V. parvula* were grown in anaerobic conditions (10% hydrogen, 10% CO_2_, and 80% nitrogen), *L. mirabilis* was grown aerobically, and the other five bacteria were grown in 5% CO_2._ All strains were grown at 37°C.

### Cell Lines and Growth Conditions

OSCC cell lines SCC4 (RRID: CVCL_1684), SCC25 (RRID: CVCL_1682), and CAL27 (RRID: CVCL_1107), in addition to telomerase immortalized human gingival keratinocytes (TIGKs; RRID: CVCL_M095) as non-cancer control, were obtained from ATCC, with certificates confirming the cells were mycoplasma-free and authenticated by STR. Dulbecco's modified Eagle's medium (DMEM) supplemented with 10% fetal bovine serum (FBS) and 2.5 mM L-glutamine was used for growing CAL27cell line. A 1:1 mixture of DMEM and Ham's F12 medium containing 2.5 mM L-glutamine, 400 ng/ml hydrocortisone and 10% FBS was used for growing cell lines SCC4 and SCC25. Dermal Cell Basal Medium (ATCC PCS-200-030) supplemented with keratinocyte growth kit (ATCC PCS-200-040) was used for growing TIGKs cells. All the cell lines were grown in 5% CO_2_ at 37°C.

### Experimental Design

The study involved testing each of the 7 bacterial strains against each of the 3 cell lines as a lysate at 3 concentrations or in co-culture at 3 multiplicity of infections (MOIs). Parameters assessed were proliferation (counting and ATP assay) and expression of 6 marker genes. All experiments were performed in triplicates for each assay and timepoint. Strains that showed prominent results in the proliferation assay were also tested on non-cancer cell line TIGKs. A total of 1,872 counts, 1,682 ATP assays, 504 RNA extractions, and 3, 982 real-time PCR reactions were performed.

### Treatment of OSCC Cells With Bacterial Lysates

Lysates were prepared from the bacterial strains grown to mid-log stage as described in Supplementary Method 1. For the proliferation assay, OSCC cells were suspended in the respective culture media supplemented with penicillin/streptomycin mix at a final concentration of 100 I.U./ml and seeded to 48 well plates (TPP, Switzerland) at a density of 7,500 cells/well. For studying gene expression, cells were seeded at 25,000–35,000 cells/well depending on the cell line. The cells were allowed to attach for 24 h before the lysates were added to a final concentration of 200, 100, or 50 μg/ml (15 mM HEPES buffer was added to the control wells). Proliferation was assessed at 24, 48, and 72 h after addition of the lysates, while RNA extraction for gene expression analysis was done at 24 h of exposure (at which time the cells were ~90% confluent).

### Co-culture of OSCC Cells With Bacteria

The bacterial strains were grown to mid log stage as described in Supplementary Method 2. Actively growing bacterial cells were pelleted by centrifugation at 5,000 rpm for 5 min, washed three times in phosphate-buffered saline (PBS), and suspended in DMEM media supplemented with 10% fetal bovine serum (FBS) and 2.5 mM L-glutamine. They were then added at MOIs of 10, 50, and 100 to the OSCC cells seeded 24 h before as described above (DMEM media added to control wells). To allow bacterial exposure throughout the experimental period without overgrowing, penicillin/streptomycin mix was included in the culture medium at sub-minimum inhibitory concentration (sub-MIC) which was determined for each bacterium prior to the experiment as described in Supplementary Method 3. Proliferation and gene expression were assessed at the same timepoints as for the lysates experiment.

### Cell Proliferation

This was assessed by cell counting and ATP assay. For counting, cells were harvested from wells using Trypsin-EDTA solution after washing with PBS. After neutralizing trypsin, harvested cells were counted using a Luna-II^TM^ automated cell counter (Logosbio). ATP was measured using the CellTiter-Glo®2.0 Cell Viability Assay Kit (Promega, USA) according to manufacturer's instructions, and luminescence was measured on a multimode plate reader (Biotek, Synergy HTX).

### RNA Extraction and Gene Expression Assays

The culture medium was removed and stored at −80°C for protein analysis, the cells were washed with PBS, and RNA was isolated using the PureLink RNA extraction kit (Invitrogen, USA) following manufacturer's instructions. SUPERase·In^TM^ RNase inhibitor (Invitrogen) was added to the extracted RNA at a concentration of 1U/μl of RNA and contaminating DNA was digested using the Turbo DNA-free^TM^ kit (Invitrogen). RNA yield and quality were assessed using a Nanodrop (ThermoFisher Scientific, USA). Extracted RNA was directly used in TaqMan® Array Standard Plates (4413266, ThermoFisher Scientific, USA) with predesigned primers/probe sets for human genes TNF-α, IL6, CD36, MMP3, CCND1, NANOG, and CASC3, employing a one-step, quantitative, reverse-transcription PCR (one-step q-RT-PCR). Specifically, the SuperScript™ III Platinum™ One-Step qRT-PCR Kit (ThermoFisher Scientific, USA) was used in 20 μl reactions as recommended by the manufacturer, using ~35 ng total RNA as template. Quantification was performed on a Quantstudio TM 3.0 Real-Time PCR system (Applied Biosystems, USA) using the following thermal cycling conditions: cDNA synthesis for 15 min at 50°C, initial denaturation at 95°C for 2 min and 40 cycles of denaturation at 90°C for 15 s and extension at 60°C for 1 min with data collection at the end of each extension step. Results were analyzed on QuantStudio 3.0 software using CASC3 gene as internal control. The gene expression levels for each target gene were calculated relative to the negative control (no exposure) using the 2^(delta delta Ct)^ method (Livak and Schmittgen, 2001). The genes' assay manufacturer IDs are shown in Supplementary Method 4.

### Statistical Analyses

For the proliferation assays (counting and ATP), two-way analysis of variance (ANOVA) with time and concentration as variables, followed by Dunnett's multiple comparison (Dunnett, 1955) was used to determine statistical significance of differences between the exposed and control cells. Significance in differences in gene expression and protein levels was sought using multiple *t-*tests comparing each concentration against the control followed by Benjamini et al. (2006) method to control for false discovery rates (FDR). GraphPad Prism 7 was used for statistical analysis and generating the figures.

## Results

### Effects on Proliferation—General Findings

The effect of bacterial lysates on proliferation of the three cell lines as assessed by counting and ATP assay is shown in Supplementary Figures 1–6. With the exception of *S. mitis*, the lysates resulted in no to marginal reduction of proliferation, with SCC4 being the least sensitive. Although the lysates from *H. parainfluenzae and N. flavescens* showed a more prominent effect on cell counts (especially in SCC25), that was not confirmed by the ATP assay. The results from the co-culture experiments are presented in Supplementary Figures 7–12. The pathogenic control *P. gingivalis* effect varied from slight decrease to slight increase in proliferation depending on the cell line and nature of exposure (lysate vs. coculture). Only *S. mitis, H. parainfluenzae and N. flavescens* resulted in pronounced reduction of proliferation, confirmed by both counting and ATP measurement. These are described in details in the following sections.

### Cytotoxicity of *S. mitis* Is Mediated by H_2_O_2_

**Figure 1:**
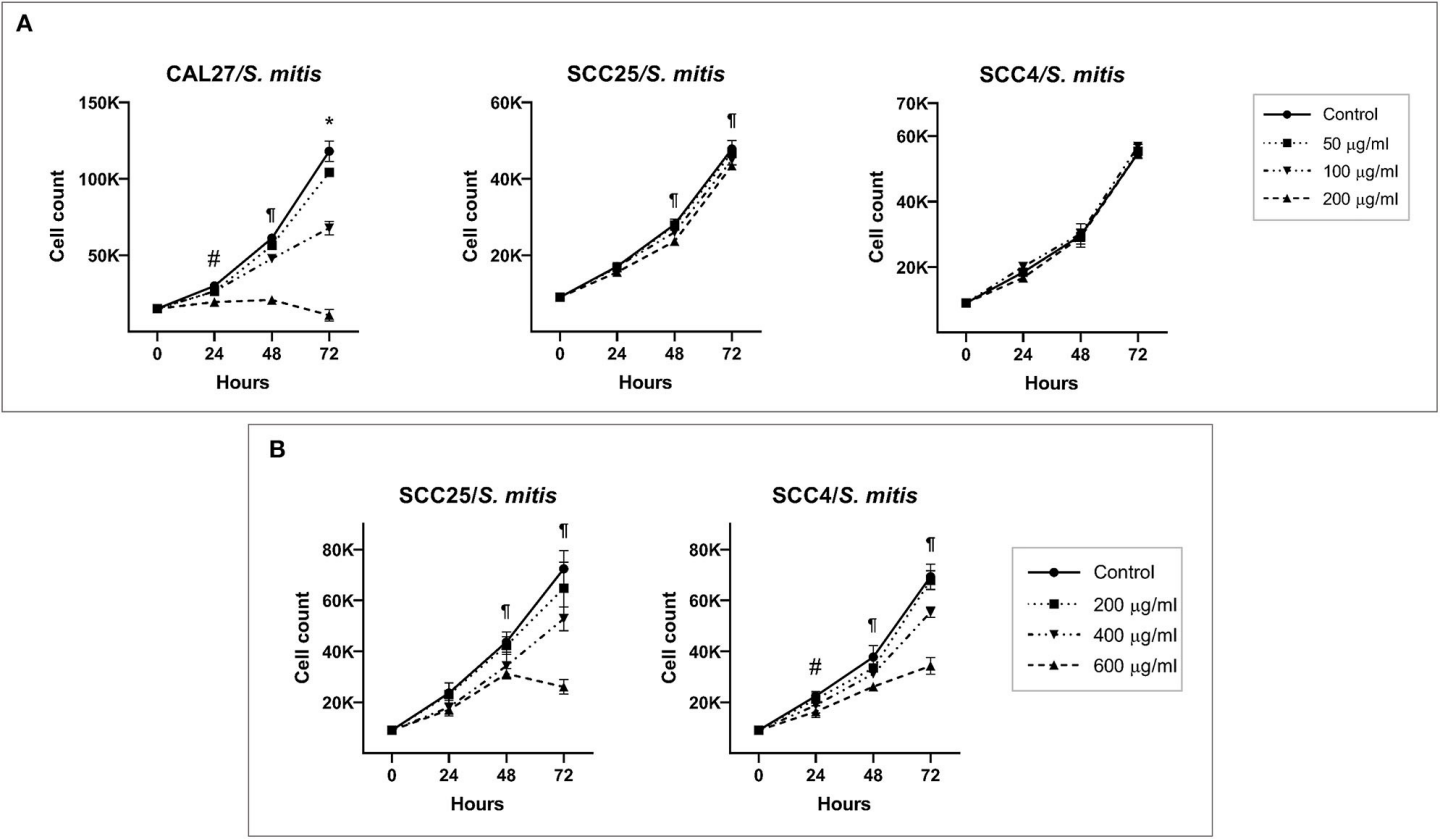
Effect of *S. mitis* lysate on proliferation of oral cancer cell lines. **(A)** CAL27, SCC25, and SCC4 were exposed to 50, 100, and 200 μg/ml of the lysate and counting performed at 24, 48, and 72 h. *Statistically significant (*p* ≤ 0.05) for all concentrations compared to the control; ^¶^for 100 and 200 μg/ml; ^#^for 200 μg/ml only. **(B)** SCC25 and SCC4 were treated with higher concentrations (400 and 600 μg/ml) of the lysates. ^¶^Statistically significant (*p* ≤ 0.05) for 400 and 600 μg/ml compared to the control; ^#^for 600 μg/ml only. The corresponding results from the ATP assay are shown in Supplementary Figures 4–6.

**Figure 2:**
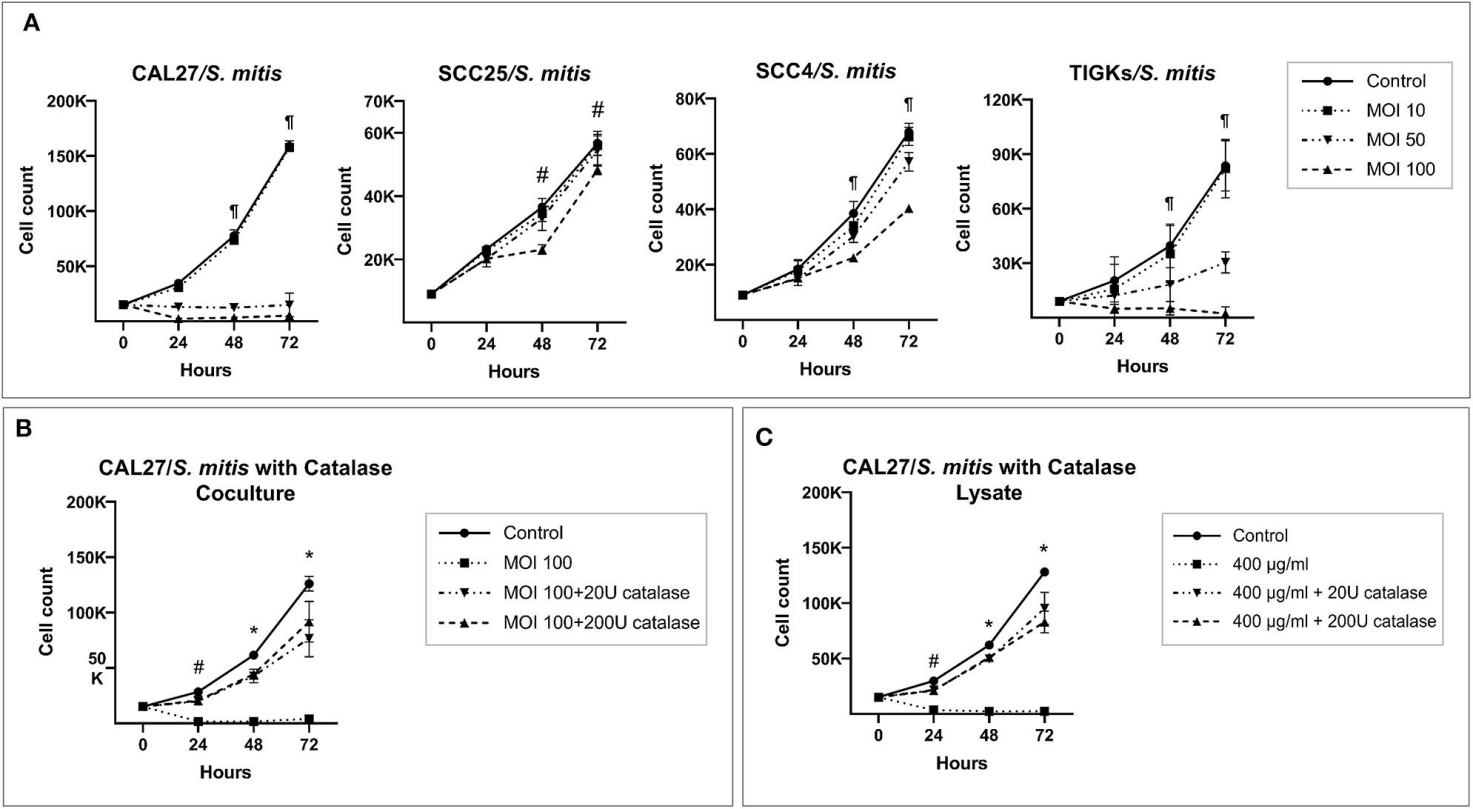
Catalase inhibits cytotoxicity of *S. mitis* against oral cancer cell lines. **(A)** CAL27, SCC25, SCC4, and TIGKs (as non-cancer control) were cocultured with *S. mitis* at MOIs of 10, 50, and 100 and counting performed at 24, 48, and 72 h. ^¶^Statistically significant (*p* ≤ 0.05) for MOI 50 and 100 compared to the control; ^#^for MOI 100 only. **(B)** CAL27 cocultured with *S. mitis* at MOI 100 in the presence or absence of exogenous catalase at 20 or 200 U. *Statistically significant (*p* ≤ 0.05) for all treatments compared to the control; ^#^for MOI 100 treatment without catalase. **(C)** Same as for **(B)** but using the lysate at 400 μg/ml instead. *Statistically significant for all treatments compared to the control; ^#^for 400 μg/ml lysate treatment without catalase.

*S. mitis* lysate led to inhibition of proliferation of CAL27 in dose-dependent manner (Figure 1A); at the highest concentration (200 μg/ml), the counts at 72 h were lower than baseline, indicating the observed effect was due to cytotoxicity. Proliferation of SCC4 and SCC25 was not affected at similar concentrations, however, treatment with higher concentrations (400 and 600 μg/ml) significantly reduced proliferation, but still not to levels seen in CAL27 (Figure 1B). In coculture, *S. mitis* reduced proliferation of all the three OSCC cell lines, but as with the lysates, CAL27 was the most sensitive (Figure 2A). Proliferation of TIGKs was affected similar to CAL27. To test if other *Streptococci* have similar properties, we cocultured CAL27 with *Streptococcus mutans* and *Streptococcus sanguinis*, and found the latter, but not the former, to result in a reduction in proliferation similar to that induced by *S. mitis* (Supplementary Figure 13). *S. sanguinis* and *S. mitis* belong the same group of Streptococci which are known produce H_2_O_2._ To find out if the cytotoxic effect of *S. mitis* observed was mediated by H_2_O_2_, we performed a separate set of experiments where 20 or 200 U/ml of Catalase (Sigma-Aldrich), an enzyme that decomposes H_2_O_2_, was added to CAL27 prior to treatment with *S. mitis* lysate at a concentration of 400 μg/ml or coculture at MOI 100. The addition of catalase inhibited *S. mitis* cytotoxicity (Figures 2B,C) although not entirely, which demonstrated that most *S mitis* cytotoxicity was mediated by H_2_O_2_. To explore the underlying mechanism further, we assessed apoptosis using the Caspase-Glo® 3/7 and RealTime-Glo™ Annexin V Apoptosis and Necrosis assays (Promega, USA), and found exposure to *S mitis* at MOI 10 and 50 induces increased caspase activity and loss if cell membrane integrity at 5 and 20 h, respectively (Supplementary Tables 1, 2).

### *H. parainfluenzae* and *N. flavescens* Inhibit Proliferation by Intracellular Infection

**Figure 3:**
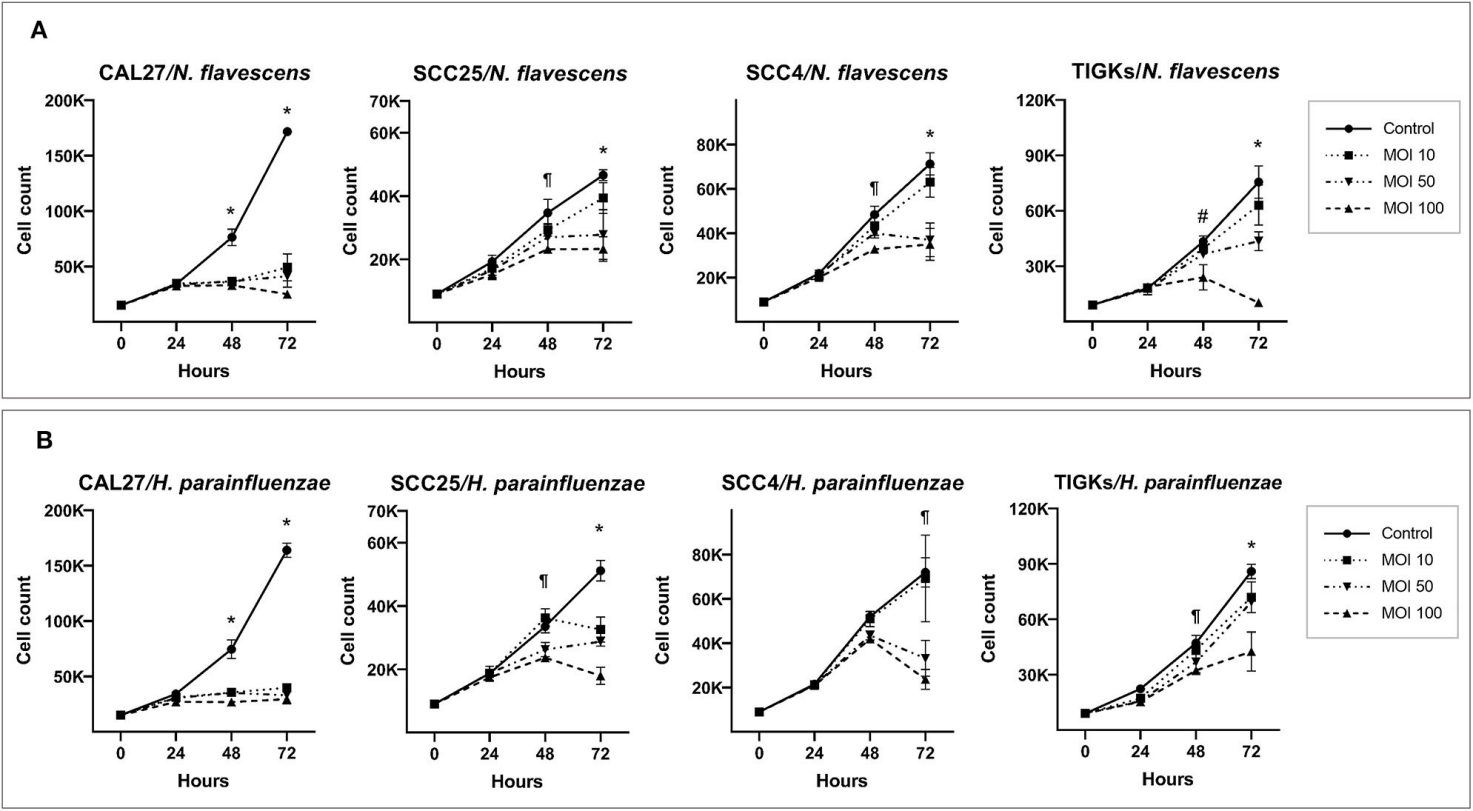
*H. parainfluenzae* and *N. flavescens* cytotoxicity against oral cancer cell lines. CAL27, SCC25, SCC4, and TIGKs (as non-cancer control) were cocultured with **(A)** *N. flavescens* and **(B)** *H. parainfluenzae* at MOIs of 10, 50, and 100 and counting performed at 24, 48, and 72 h. *Statistically significant (*p* ≤ 0.05) for all MOIs against the control; ^¶^for MOI 50 and 100; ^#^for MOI 100 only. The corresponding results from the ATP assay are shown in Supplementary Figures 10–12.

**Figure 4:**
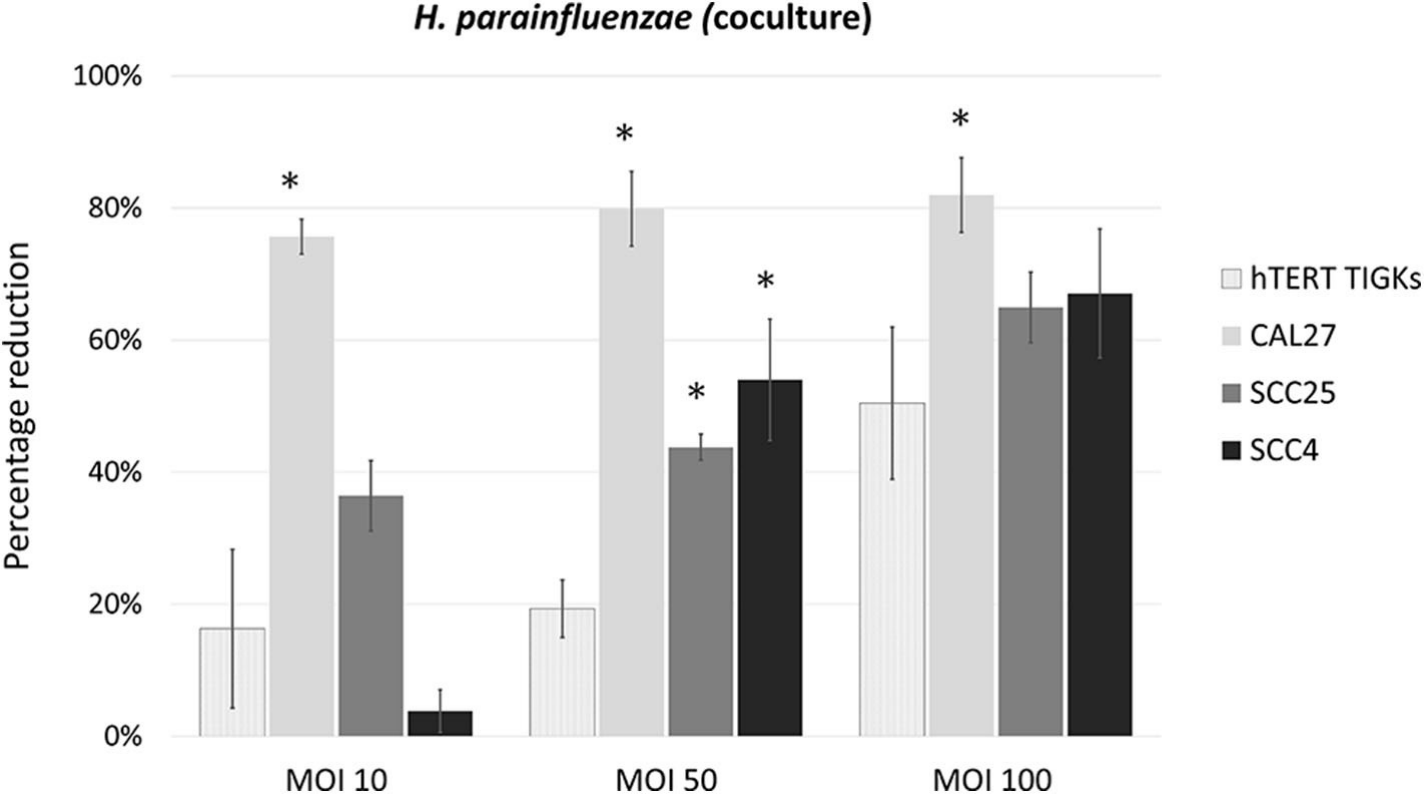
Percentage reduction of cell proliferation by *H. parainfluenzae* in CAL27, SCC25, SCC4 and TIGKs in cocultures. *Statistically significant (*p* ≤ 0.05) compared to TIGKs; pair-wise Student's *t-*test.

*H. parainfluenzae* and *N. flavescens* caused substantial inhibition of cell proliferation in all the three OSCC cell lines as well as TIGKs in a dose-dependent manner, with CAL27 being the most sensitive (Figure 3). For *S. mitis* and *N. flavescens*, reduction in proliferation of TIGKs did not significantly differ from that of the OSCC cell lines (Supplementary Figure 14); however, *H. parainfluenzae* resulted in significantly higher inhibition of OSCC cell lines compared to TIGKs (Figure 4), especially at MOI 50. Interestingly, *N. flavascens* and *H. parainfluenzae* in coculture with the cell lines showed strong growth after 48 h of inoculation despite presence of sub-MIC of antibiotics that was otherwise sufficient to control growth in the absence of the cells (results shown for *N. flavascens* with CAL27 and SCC4 in Supplementary Tables 3, 4), which was taken as an evidence of intracellular infection. To confirm that, we performed intracellular vs. extracellular bacteria staining as described in Supplementary Method 5. Images obtained (Supplementary Figures 15–17) revealed internalization of both bacteria in the three cell lines. This was further confirmed by performing antibiotic protection assays (Supplementary Method 6). The numbers of viable intracellular bacteria recovered from each of the three cell lines are shown in Supplementary Tables 5, 6.

### Effect on Gene Expression—General Findings

Changes in the expression of the six marker genes in response to exposure to the bacterial lysates are shown for the three cell lines in Supplementary Figures 18–20, while the corresponding results from the co-culture experiments are illustrated in Supplementary Figures 21–23. A fold change of ≥2 was used to demarcate pronounced effects. Each cell line showed a unique response. Some changes in gene expression were concentration-dependent while others were concentration-independent. Overall, the results from the lysate and co-culture experiments were consistent. CCND1 was the least responsive gene in all cell lines, followed by NANOG. Results for MMP3 varied significantly by cell line and the nature of exposure (lysate vs. coculture). More consistent and profound results, which are explained in more detail below, were observed for CD36, IL6, and TNF-α.

### CD36 Gene Expression Down-Regulated by Health Associated Species

**Figure 5:**
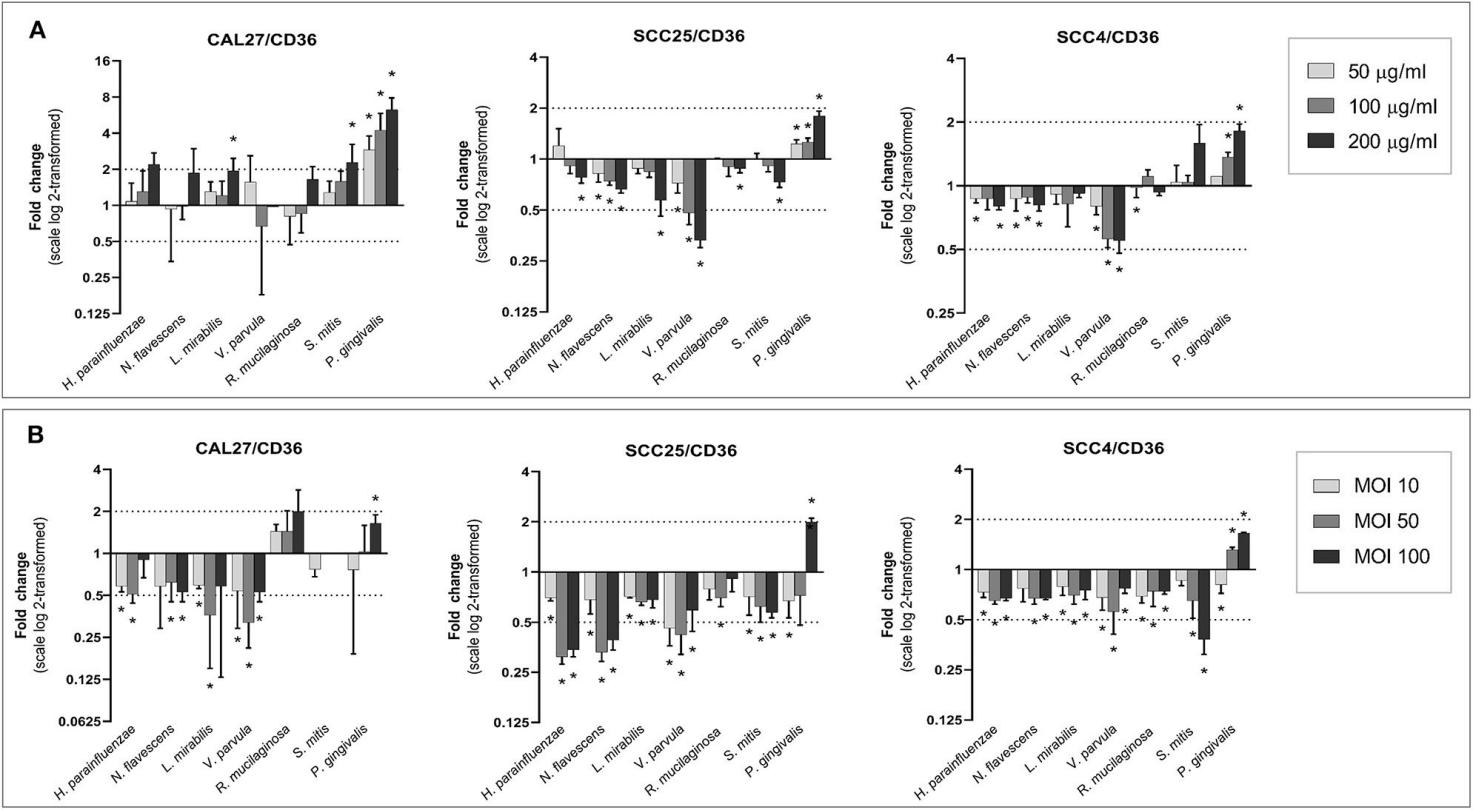
Effect of the test bacteria on expression of CD36. CAL27, SCC25, and SCC4 were either **(A)** exposed to 50, 100, and 200 μg/ml of each of the bacterial lysates, or **(B)** cocultured with each bacterium at MOIs of 10, 50, and 100. RNA was extracted after 24 h and CD36 was quantified using one-step q-RT-PCR, using CASC3 as reference gene. *Statistically significant (*p* ≤ 0.05) compared to the non-treated control. Dashed line above and below horizontal axis indicate a 2-fold change.

The effect of different bacteria on the expression of CD36 is shown in Figure 5. Lysates from the Gram-negative, health-associated bacteria, particularly *V. parvula*, caused a downregulation of CD36 in SCC25 and SCC4, whereas the lysate of *P. gingivalis* led to an upregulation in all cell lines, prominently in CAL27. The effects were mostly dose-dependent. In cocultures, all health-associated species resulted in downregulation of CD36 in all cell lines (with the exception of *R. mucilaginosa* in CAL27); in most cases, the effect peaked at MOI 50. The effect of *P. gingivalis* varied with concentration, with the lowest MOI resulting in downregulation while the highest concentration associated with upregulation of CD36.

### Universal Upregulation of IL6 and TNF-α

**Figure 6:**
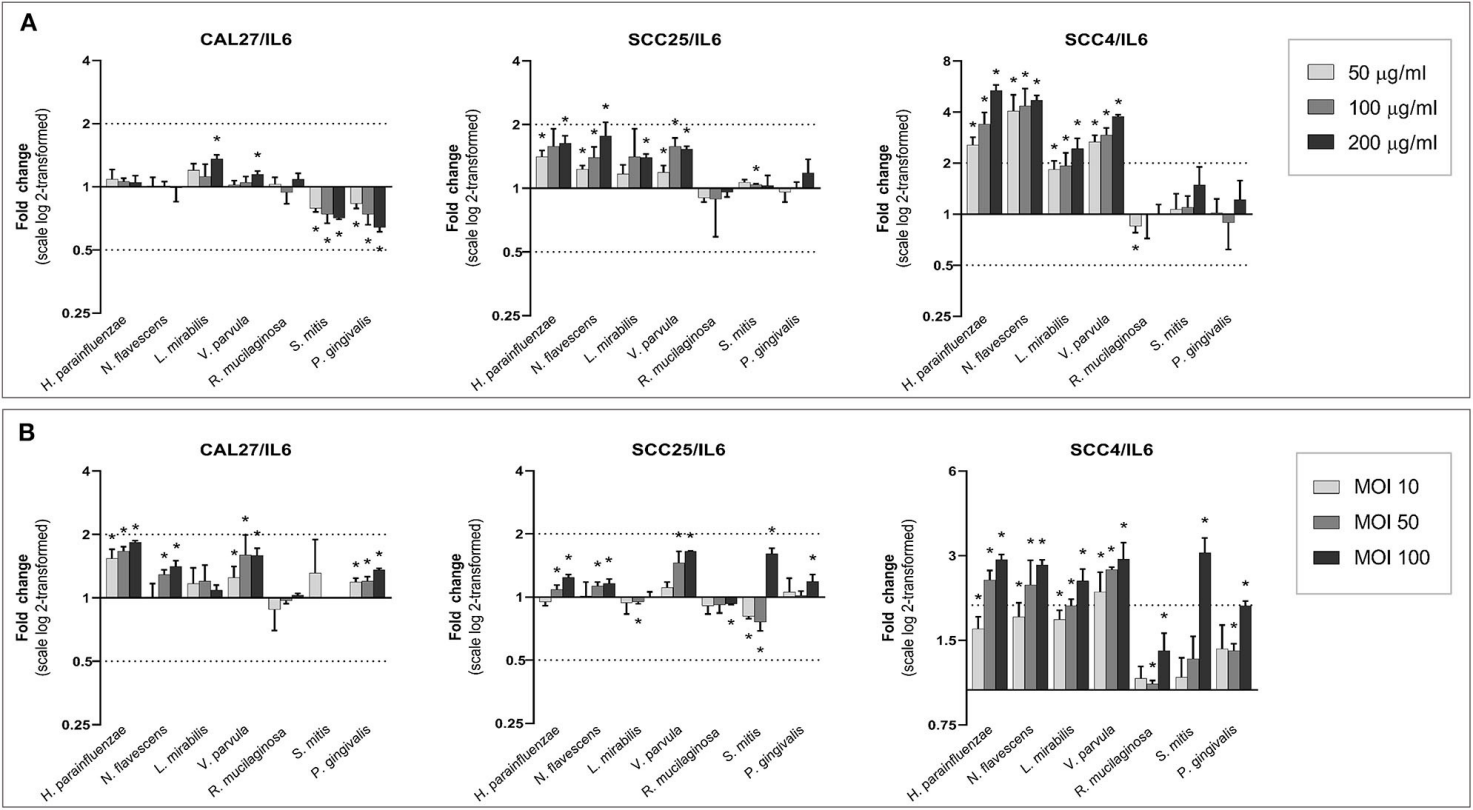
Effect of the test bacteria on expression of IL6. CAL27, SCC25, and SCC4 were either **(A)** exposed to 50, 100, and 200 μg/ml of each of the bacterial lysates, or **(B)** cocultured with each bacterium at MOIs of 10, 50, and 100. RNA was extracted after 24 h and IL6 was quantified using one-step q-RT-PCR, using CASC3 as reference gene. *Statistically significantly compared to the non-treated control. Dashed line above and below horizontal axis indicate a 2-fold change.

**Figure 7:**
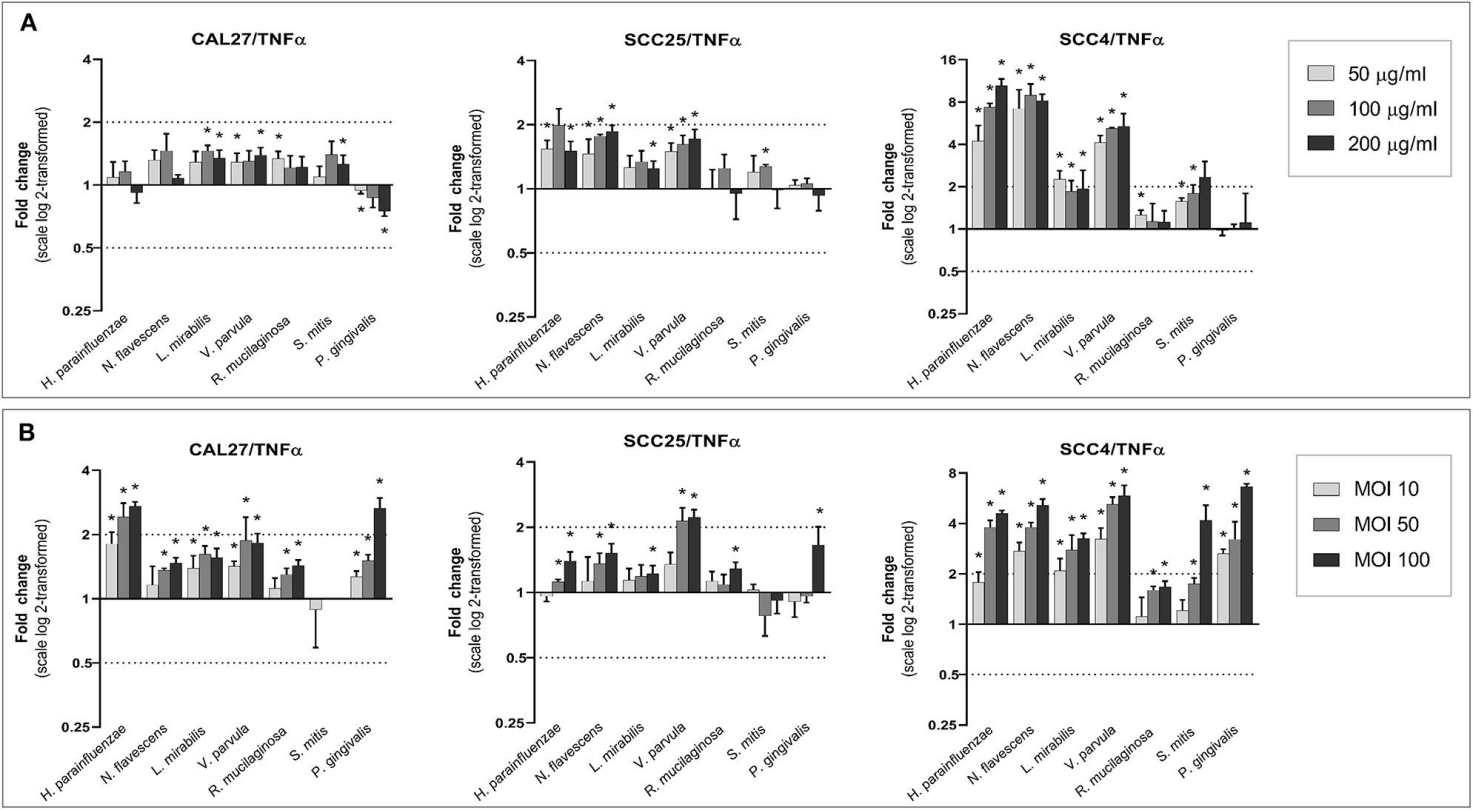
Effect of the test bacteria on expression of TNFα. CAL27, SCC25, and SCC4 were either **(A)** exposed to 50, 100, and 200 μg/ml of each of the bacterial lysates, or **(B)** cocultured with each bacterium at MOIs of 10, 50, and 100. RNA was extracted after 24 h and TNFα was quantified using one-step q-RT-PCR, using CASC3 as reference gene. *Statistically significant compared to the non-treated control. Dashed line above and below horizontal axis indicate a 2-fold change.

With a few exceptions, all species, particularly the Gram-negatives, increased the expression of IL6 and TNF-α in a dose-dependent manner (Figures 6, 7). The upregulation was profound in SCC4. The effect of *H. parainfluenzae, N. flavescens* and *V. parvula* was consistent between the lysate and co-culture experiments. For *P. gingivalis* a notable upregulation was only observed in coculture, while the lysates did not result in changes in expression of the two genes in SCC4 and SCC25 and even downregulated them in CAL27. *R. mucilaginosa* upregulated TNF-α, but hardly affected the expression of IL6. For *S. mitis*, the results varied by cell line and from the lysates to co-culture. To validate the results, we performed protein analysis of IL6 and TNF-α in supernatants from all the cell lines co-cultured with *H. parainfluenzae, N. flavescens, S. mitis*, and *P. gingivalis* at MOI 100 (Supplementary Method 7). We found the changes in protein levels to correlate with those identified by gene expression except for P. gingivalis (Supplementary Figure 24), for which IL6 and TNF-α (and all other proteins) tested low to non-detectable, probably due to break down by proteolytic enzymes produced by *P. gingivalis*.

## Discussion

The emerging role of microbiome in oral cancer is gaining much attention. The carcinogenic properties of oral pathogens like *P. gingivalis* and *F. nucleatum* are well-documented; however, not much is known about the role of health-associated commensals in oral cancer. In this study, we have attempted to understand the *in-vitro* effects of health-associated commensals on cell proliferation and expression of select genes in different OSCC cell lines. We tested both lysates and living bacteria to assess whether any effects are a result of bacterial components or products, or a direct interaction between the bacteria and the tumor cells. For assessment of proliferation, we supplemented cell counting with ATP assay to improve reliability. In the co-culture experiments, we employed a novel approach in which sub-MIC concentration of antibiotics was used to allow bacterial cells to remain viable but without overgrowing. The purpose was to have the bacteria interact with cells for the entire duration of experiment (72 and 24 h for the proliferation and gene expression assays, respectively). Previous studies typically used short exposure time (45 min to 2 h) to circumvent bacterial overgrowth in similar experiments (Hasegawa et al., 2007; Boonanantanasarn et al., 2012; Okahashi et al., 2014). Nevertheless, it is possible that antibiotics, even at sub-MIC levels, could have affected the bacterial cell wall structure and protein synthesis, which in turn, may explain, at least in part the differences in the results obtained with living cells and cell lysates.

Three species resulted in significant reduction of proliferation: *S. mitis, N. flavescens* and *H. parainfluenzae*. For *S. mitis*, the effects were observed with both the lysate as well as in co-culture. Several studies have shown it to be depleted in OSCC patients (Pushalkar et al., 2012; Schmidt et al., 2014; Al-Hebshi et al., 2017; Amer et al., 2017), and to negatively correlate with tumor staging (Yang et al., 2018), suggesting it may be playing a protective role against oral cancer. The cytotoxic effect observed in this study was inhibited by addition of catalase, indicating it was mediated by H_2_O_2._ This is not an entirely novel finding, given that the closely related species *Streptococcus oralis* and *Streptococcus sanguinis* have previously been shown to induce H_2_O_2_-mediated cytotoxicity in macrophages and epithelial cells (Okahashi et al., 2011, 2013, 2014). The addition of catalase, however, did not completely reverse the cytotoxic effects, suggesting that part of the cytotoxicity seen is H_2_O_2−_independent. Okahashi et al. (2014) observed the same for *S. oralis*. In contrast, and in total contradiction with these findings, Boonanantanasarn et al. (2012) demonstrated H_2_O_2_ produced by *E. faecalis* to enhance proliferation of oral cancer cell lines. One of the major reasons that can account for this drastic difference, is that the exposure of cells to bacteria and H_2_O_2_ in the latter study was limited to 45 min, which is brief compared to our study.

H_2_O_2_ induces oxidative stress in mammalian cells which signals the activation of several cellular pathways including of pro-inflammatory cytokine production and cell death or apoptosis (Pelaia et al., 2004; Valko et al., 2007; Forman et al., 2010; Vilema-Enriquez et al., 2016; Park, 2018; Aggarwal et al., 2019). H_2_O_2_ acts as double-edged sword; while it is shown to be a potent carcinogen in normal cells (Lisanti et al., 2011), localized production of H_2_O_2_ has been used in hyperoxygenation therapy for cancer treatment (Liu and Wang, 2015; Vilema-Enriquez et al., 2016; Mast and Kuppusamy, 2018; Perillo et al., 2020). Studies have also shown that cancer cells are more sensitive to H_2_O_2_ than normal cells (Aykin-Burns et al., 2009) which has led to increased interest in exploring its therapeutic potential for cancer treatment including oral cancer. S. *mitis* is a core oral bacterial species, so the relevance of H_2_O_2_ it produces, along with related species, to oral cancer, or protection thereof, is worth further investigation.

*Neisseria* and *Haemophilus* spp. are ubiquitous oral commensals that have been found in lower abundance in OSCC patients compared to healthy controls (Al-Hebshi et al., 2017; Amer et al., 2017; Guerrero-Preston et al., 2017; Perera et al., 2017; Hayes et al., 2018). The two tested species in this study*, N. flavescens* and *H. parainfluenzae*, were found to induce cytotoxicity by intracellular infection. While the genera *Neisseria* and *Haemophilus* are known to be capable of causing intracellular infection as exemplified by *Haemophilus influenzae* and Neisseria meningiditis (Nikulin et al., 2006; Parisi and Martinez, 2014), this is the first report of internalization of *N. flavescens* and *H. parainfluenzae per se*. Interestingly, *H. parainfluenzae* showed significantly less cytotoxicity against non-cancer TIGK cells as compared to OSCC cells. Whether this may play a protective role against oral cancer, or can be exploited for prevention and/or treatment of oral cancer needs further investigation, for example by extending this work to *in-vivo* mouse models of oral cancer.

In the gene expression experiments, the results from lysate and coculture were largely consistent, suggesting most of the observed effects do not involve direct interaction between the bacteria and host cell. The only exception was *P. gingivalis* for which the results substantially differed between the lysate and coculture particularly of *IL6* and *TNF-*α, that were notably upregulated only in coculture, suggesting internalization of *P. gingivalis* is important for these effects. Three genes were prominently modulated by the different species: *CD36, IL6*, and *TNF-*α. CD36 is an important transmembrane protein receptor which involved in regulating immune response, cell adhesion and lipid metabolism. CD36 plays an important role in cancer by accelerating tumor growth and metastasis, and modulating the immune response and response to therapy, and has thus been a target for cancer treatment (Wang and Li, 2019). Recently, Sakurai et al. (2020) have demonstrated the role of CD36 in facilitating the proliferation and migration activity of OSCC cells, possibly by inhibiting β-catenin signaling pathway, and they emphasized its usefulness in the diagnosis of high-grade tumor and targeted therapy of oral cancer. Interestingly, the health-associated species in this study, particularly the Gram-negatives, showed significant downregulation in *CD36* expression in contrast with the pathogenic control *P. gingivalis* that upregulated it. This is an important finding given the key role of *CD36* in cancer. The possibility of targeting it with these oral health-associated species should be explored in future studies.

*TNF-*α and *IL6* are important pro-inflammatory cytokines known to have pleotropic function in immune response, inflammation, cell death and tumor progression. In our study, significant upregulation of *TNF-*α and *IL6* was induced by both health-associated species as well as *P. gingivalis*. However, that does not necessarily mean the upregulation is associated with same biological outcomes. Both *TNF-*α and *IL6* are known to have complex effects and dual roles in cancer (Bertazza and Mocellin, 2010; Fisher et al., 2014). Some studies have shown TNF-α to possess anti-tumor properties and can be exploited for treatment of some cancer types, while others have demonstrated it contributes to tumor progression by modulation of the immune response (Montfort et al., 2019). The overall effect seems to be dependent on its level, with high concentrations favoring tumor regression. Similarly, increased expression of IL6 has been associated with tumor progression and metastasis but, at the same time, it is shown to augment adaptive immunity against tumor growth (Fisher et al., 2014). It has been emphasized that the biological outcome of these cytokines depends on multiple factors including cell type, concentration, tissue condition, tumor microenvironment, and cross talk between several signaling pathways activated at a particular time (Fisher et al., 2014; Montfort et al., 2019). We believe that further studies focusing on global transcriptome profiling are needed to get better understanding of biological mechanisms activated by commensals in OSCC cell lines.

One of the interesting findings of the study is that different OSCC lines exhibited distinct responses. This was probably due to differences in the expression of surface receptors between the three cell lines. For example, it has been shown that SCC4 has lower expression of TLR4 compared to CAL27 (He et al., 2015). The cells also have different doubling time, with CAL27 being the fastest, which probably explains it was more susceptible to the cytotoxic effects of the different bacteria.

While the bacterial species that resulted in pronounced reduction in proliferation were also tested on TIGKs as non-cancer cell control, it should be noted that immortalized cells bypass senescence similar to cancer cells, and most cancer cells also show an increased expression of hTERT proteins (Boehm and Hahn, 2004; Zhang et al., 2016). Therefore, it may have been more reliable to use primary gingival keratinocytes for assessing differential effects of the bacterial species on cancer vs. non-cancer cells. Another limitation of our study is that the expression of marker genes was restricted to only a single time point (24 h) due to logistical constraints, namely the large-scale nature of the experiments. It would have been interesting to follow the change in gene expression at also 48 and 72 h. Moreover, in addition to proliferation and gene expression of marker gene, it may have been useful to perform some assays of DNA damage and apoptosis to get more mechanistic insights, but again, that was not feasible given it was primarily a screening study.

Furthermore, considering the exploratory nature of this study, we used only six marker genes to screen the possible changes at molecular level, but there are probably many more genes that have been affected. This, however, can be addressed using a high throughput approach like RNA Seq or microarray analysis in a future study. In this *in-vitro* study, we assessed the effect of single species on each cell line, but in *in vivo* conditions, the tumor is exposed to a microbial consortium, so for future studies it may be important to use mixtures of oral bacterial species. Finally, *in vivo*, bacteria do not only interact with the tumor cells but also with their microenvironment that is known to play an important role in modulation of oral cancer, which highlights the importance of taking this work further to animal models of oral cancer.

In conclusion, this is the first study to perform large-scale, *in vitro* screening for the effect of oral health-associated bacteria on OSCC cell lines. This was required to fill in some of the knowledge gap in this area, and to form the basis and generate hypotheses for future work. Indeed, a number of interesting findings were revealed. Particularly the differential cytotoxicity by *H. parainfluenzae* against the oral cancer cell lines and the downregulation of CD36 by the health-associated species, and the possibility of exploiting them for prevention or/and treatment of oral cancer, are worth further investigation. Also, profiling of the global transcriptome rather than select genes is needed to provide a better and comprehensive understanding of how oral health-associated species may modulate the behavior of oral cancer cells.

## Data Availability Statement

The raw data supporting the conclusions of this article will be made available by the authors, without undue reservation.

## Author Contributions

NNA conceived and designed the study. DB, MY, VT, and SP assisted in the design of experiments. DB conducted and performed the majority of the experiments and data entry. VT, RL, and LV assisted with lab work and provided technical advice. NNA and DB performed data analysis. NNA, DB, and VJ drafted the manuscript. All authors read and approved the final version of the article.

## Conflict of Interest

The authors declare that the research was conducted in the absence of any commercial or financial relationships that could be construed as a potential conflict of interest.
