# Supplementary material for: Screening of Health-Associated Oral Bacteria for Anticancer Properties in vitro
Source: Front Cell Infect Microbiol. 2020 Oct 6;10:575656. doi: 10.3389/fcimb.2020.575656 (PMC7573156; doi:10.3389/fcimb.2020.575656)
Supplement: Supplementary file 1 [file Presentation_1.PDF]

Supplementary Figure 1. Effect of bacterial lysates on CAL27 proliferation (counting)

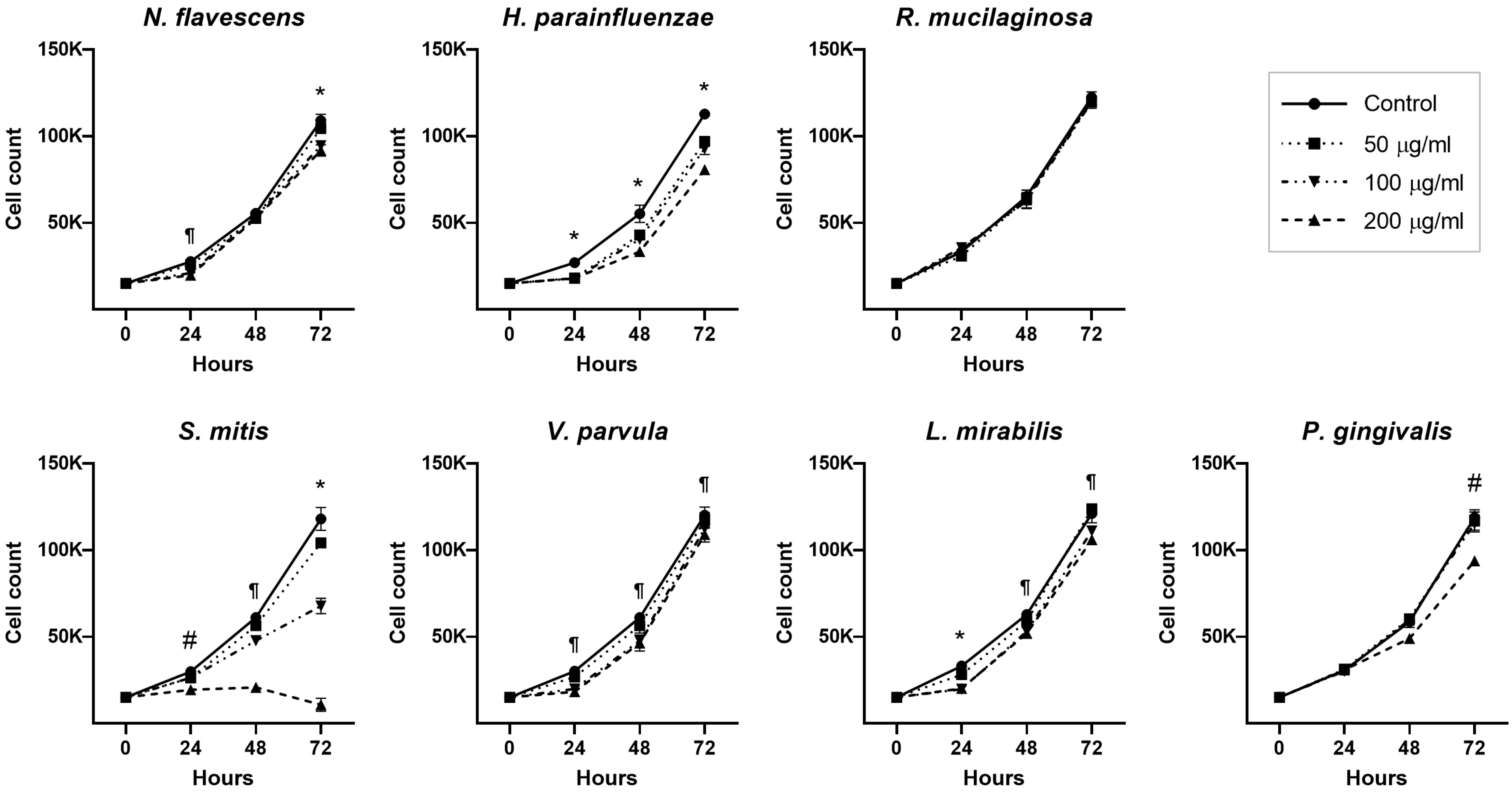

Significant difference for * all concentrations; ¶ 100 and 200 μg/ml; # 200 μg/ml; § 50 μg/ml ; ^ 100 μg/ml; v 50 and 100 μg/ml; $ 50 and 200 μg/ml

Supplementary Figure 2. Effect of bacterial lysates on SCC25 proliferation (counting)

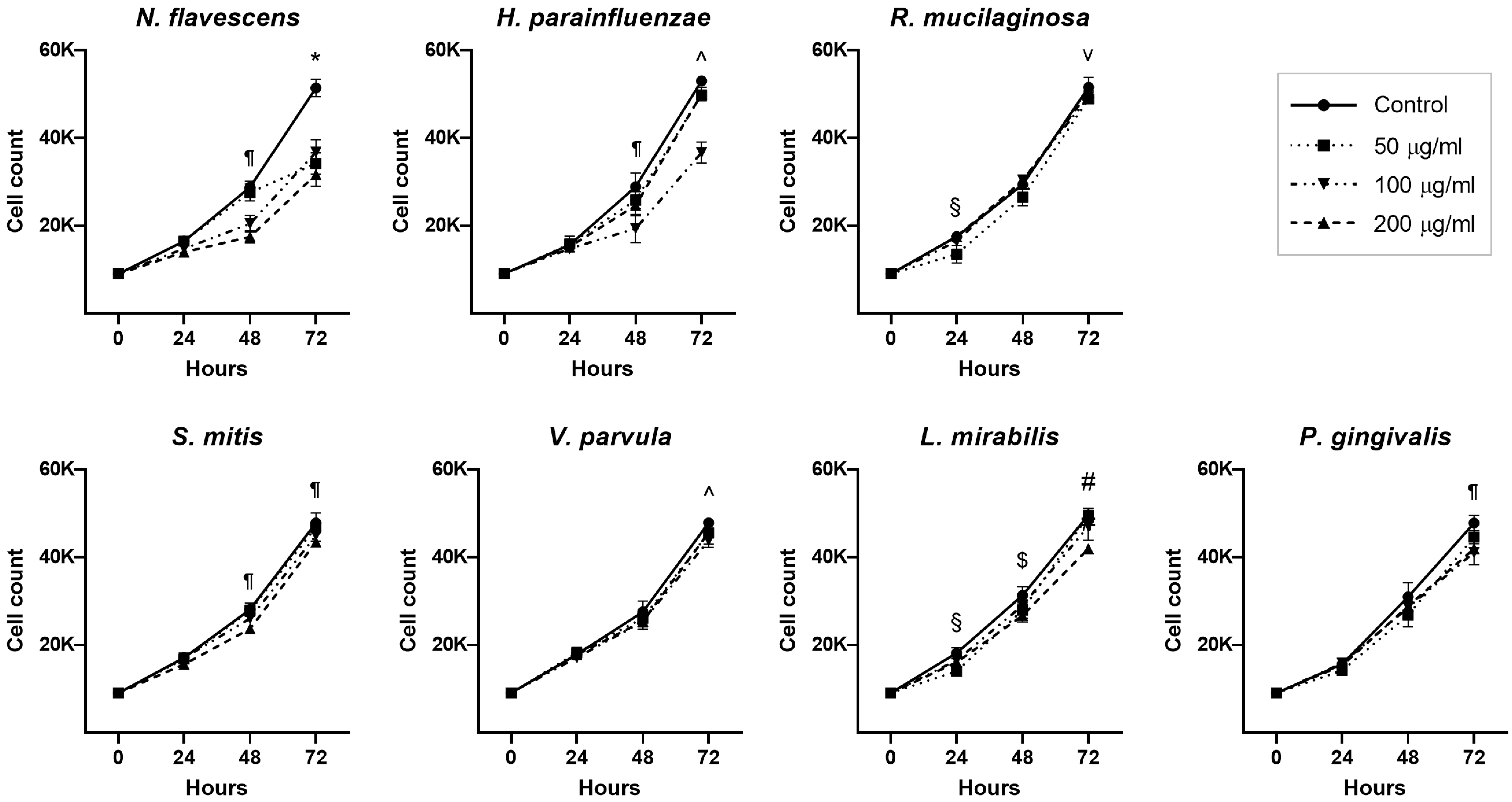

Significant difference for * all concentrations; ¶ 100 and 200 μg/ml; # 200 μg/ml; § 50 μg/ml ; ^ 100 μg/ml; v 50 and 100 μg/ml; $ 50 and 200 μg/ml

Supplementary Figure 3. Effect of bacterial lysates on SCC4 proliferation (counting)

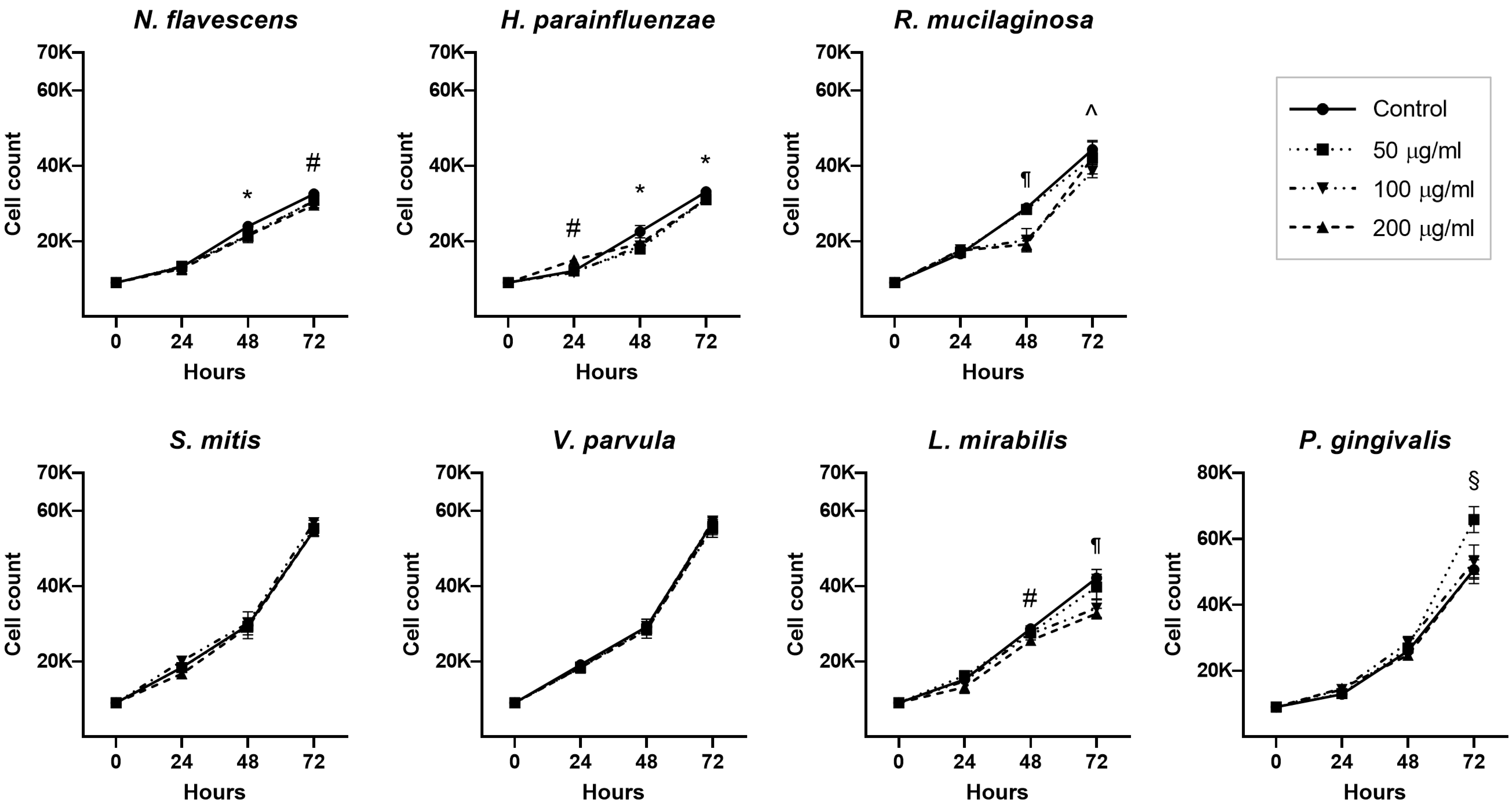

Significant difference for * all concentrations; ¶ 100 and 200 µg/ml; # 200 µg/ml; § 50 µg/ml ; ^ 100 µg/ml; v 50 and 100 µg/ml; $ 50 and 200 µg/ml

Supplementary Figure 4. Effect of bacterial lysates on CAL27 proliferation (ATP assay)

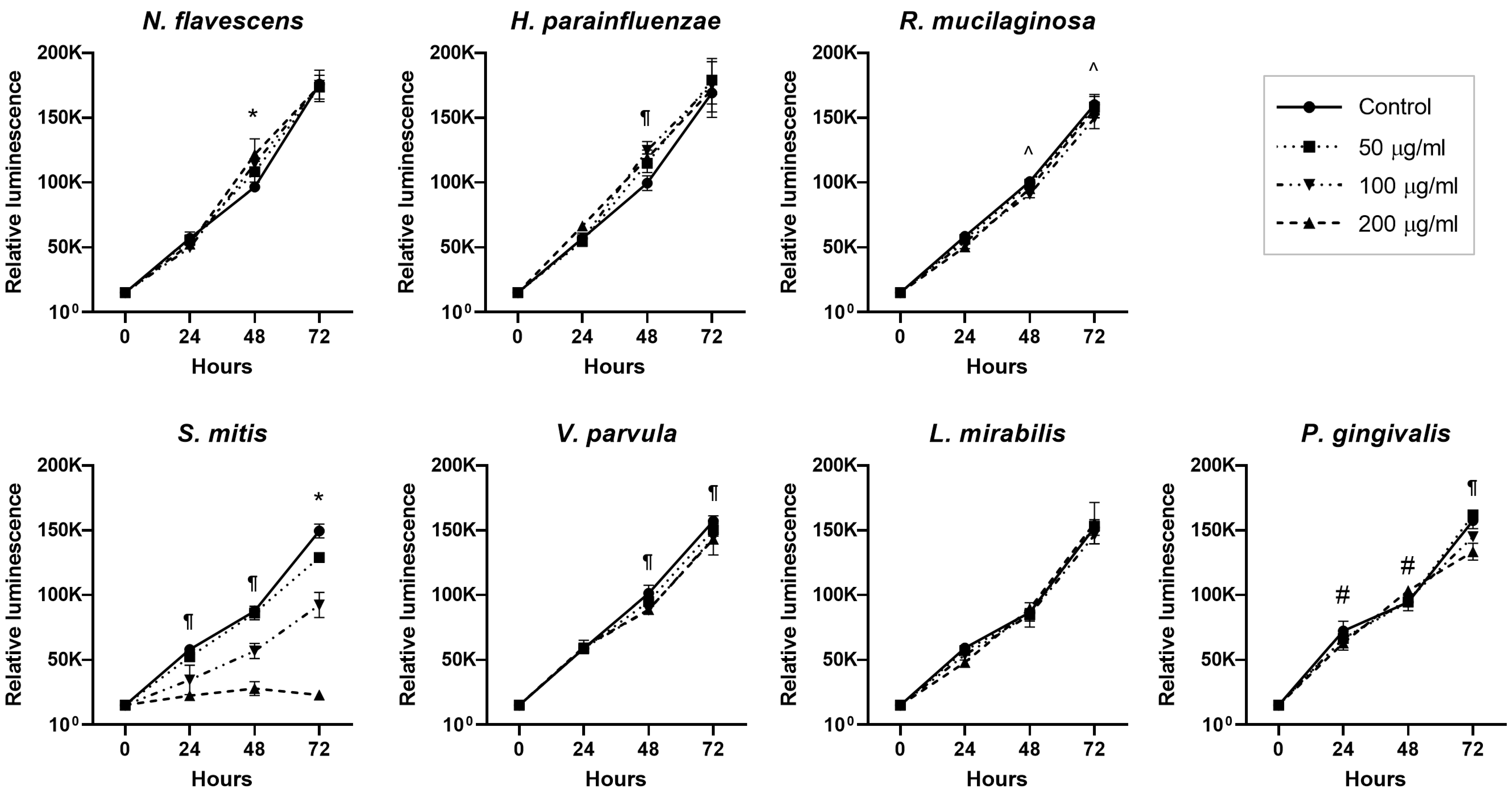

Significant difference for * all concentrations; ¶ 100 and 200 μg/ml; # 200 μg/ml; § 50 μg/ml ; ^ 100 μg/ml; v 50 and 100 μg/ml; $ 50 and 200 μg/ml

Supplementary Figure 5. Effect of bacterial lysates on SCC25 proliferation (ATP assay)

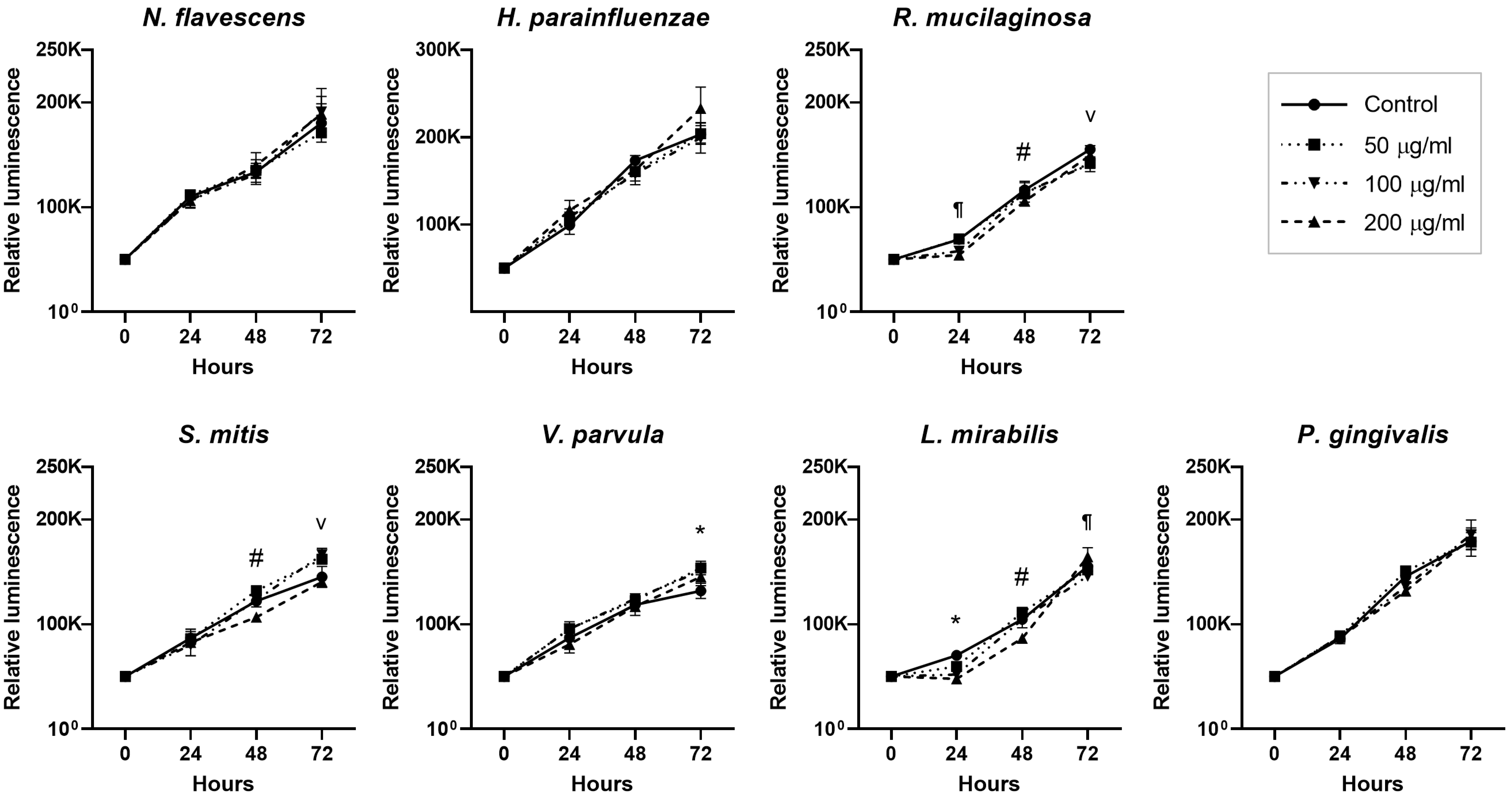

Significant difference for * all concentrations; ¶ 100 and 200 μg/ml; # 200 μg/ml; § 50 μg/ml ; ^ 100 μg/ml; v 50 and 100 μg/ml; $ 50 and 200 μg/ml

Supplementary Figure 6. Effect of bacterial lysates on SCC4 proliferation (ATP assay)

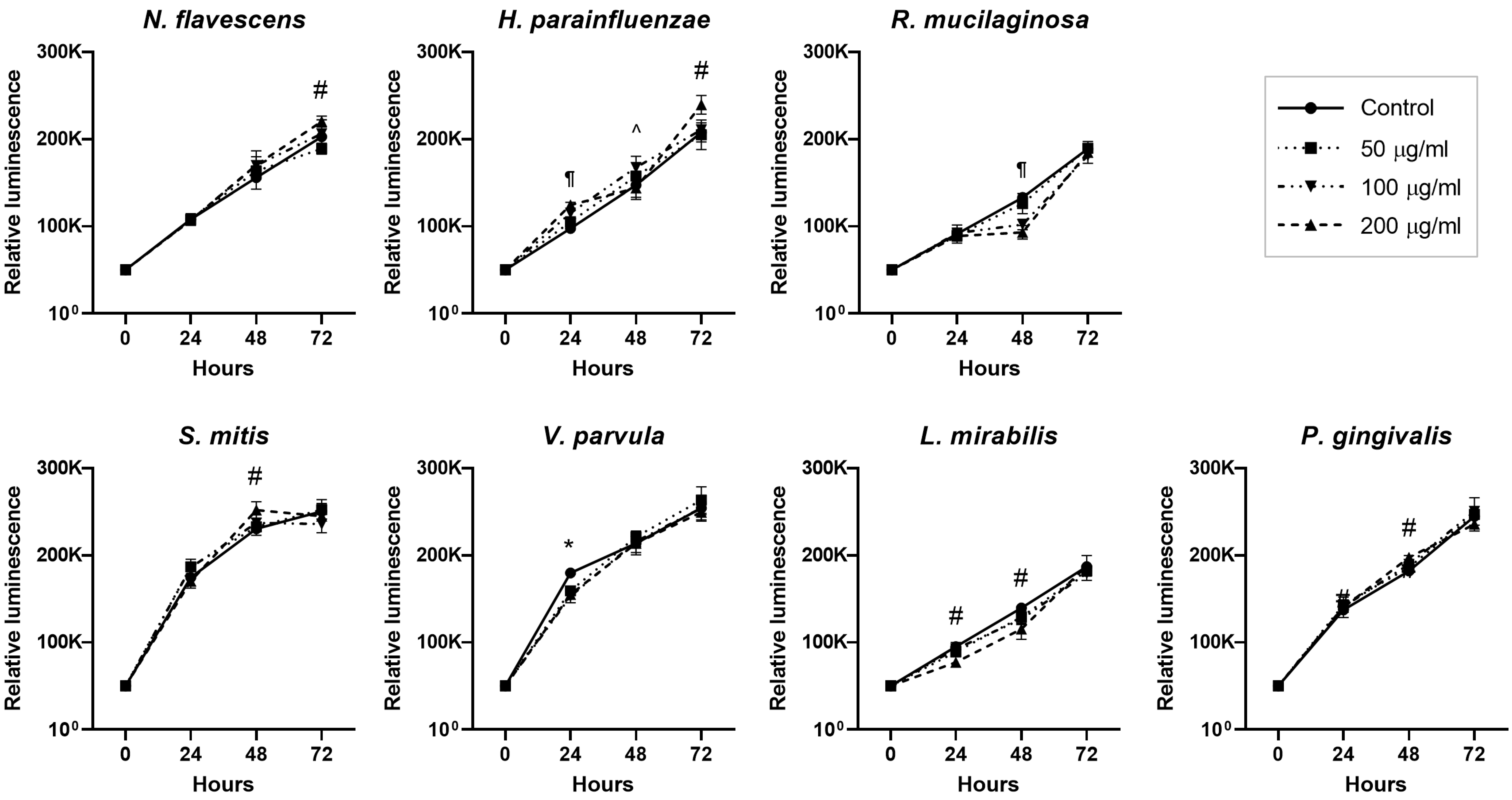

Significant difference for * all concentrations; ¶ 100 and 200 μg/ml; # 200 μg/ml; § 50 μg/ml ; ^ 100 μg/ml; v 50 and 100 μg/ml; $ 50 and 200 μg/ml

Supplementary Figure 7. Effect of cocultures with bacteria on CAL27 proliferation (counting)

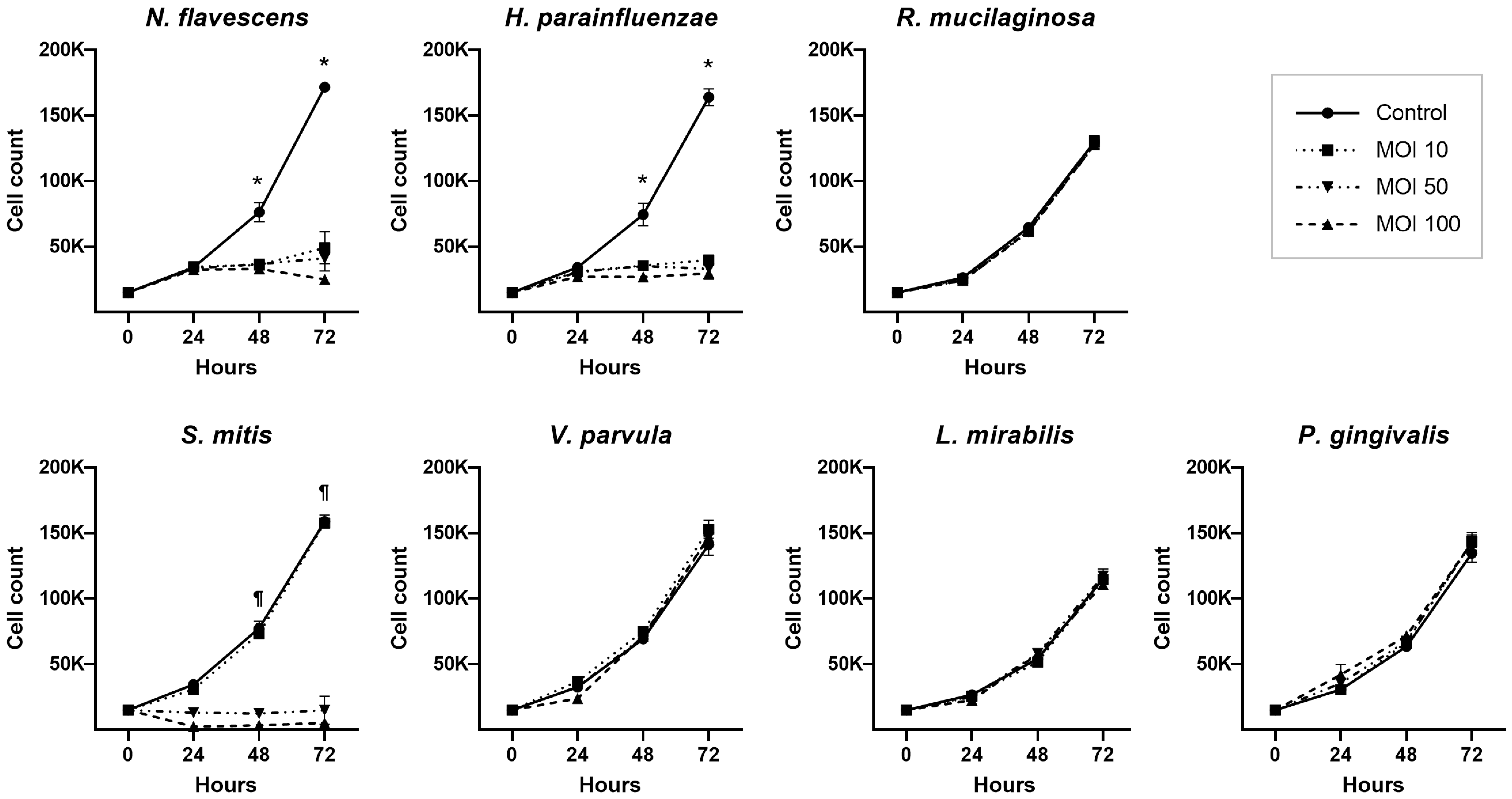

Significant difference for * all concentrations; ¶ MOI 50 and 100; # MOI 100; § MOI 10 ; ^ MOI 50; v MOI 10 and 50; $ MOI 10 and 100

Supplementary Figure 8. Effect of cocultures with bacteria on SCC25 proliferation (counting)

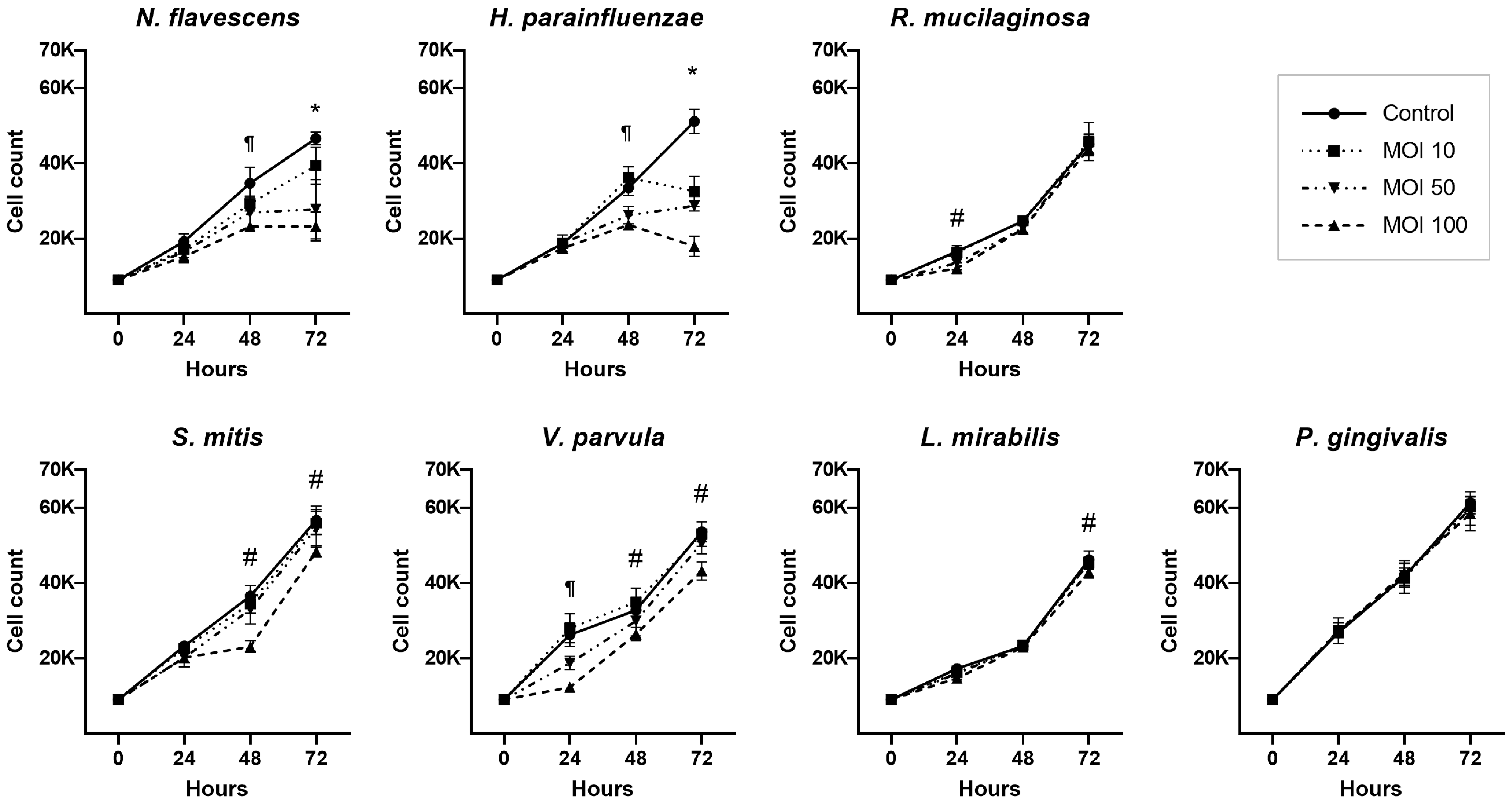

Significant difference for * all concentrations; ¶ MOI 50 and 100; # MOI 100; § MOI 10 ; ^ MOI 50; v MOI 10 and 50; $ MOI 10 and 100

Supplementary Figure 9. Effect of cocultures with bacteria on SCC4 proliferation (counting)

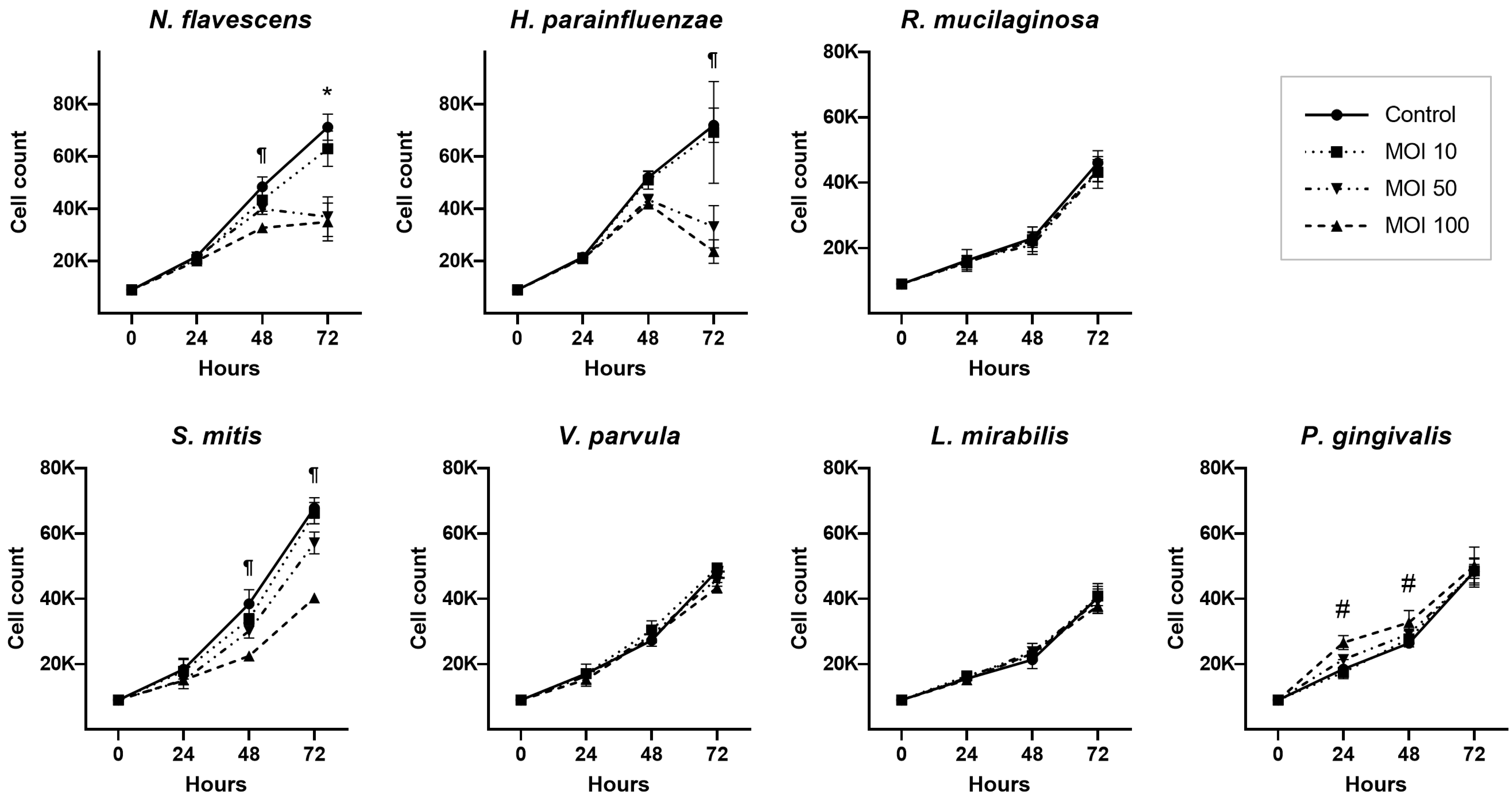

Significant difference for * all concentrations; ¶ MOI 50 and 100; # MOI 100; § MOI 10 ; ^ MOI 50; v MOI 10 and 50; $ MOI 10 and 100

Supplementary Figure 10. Effect of cocultures with bacteria on CAL27 proliferation (ATP assay)

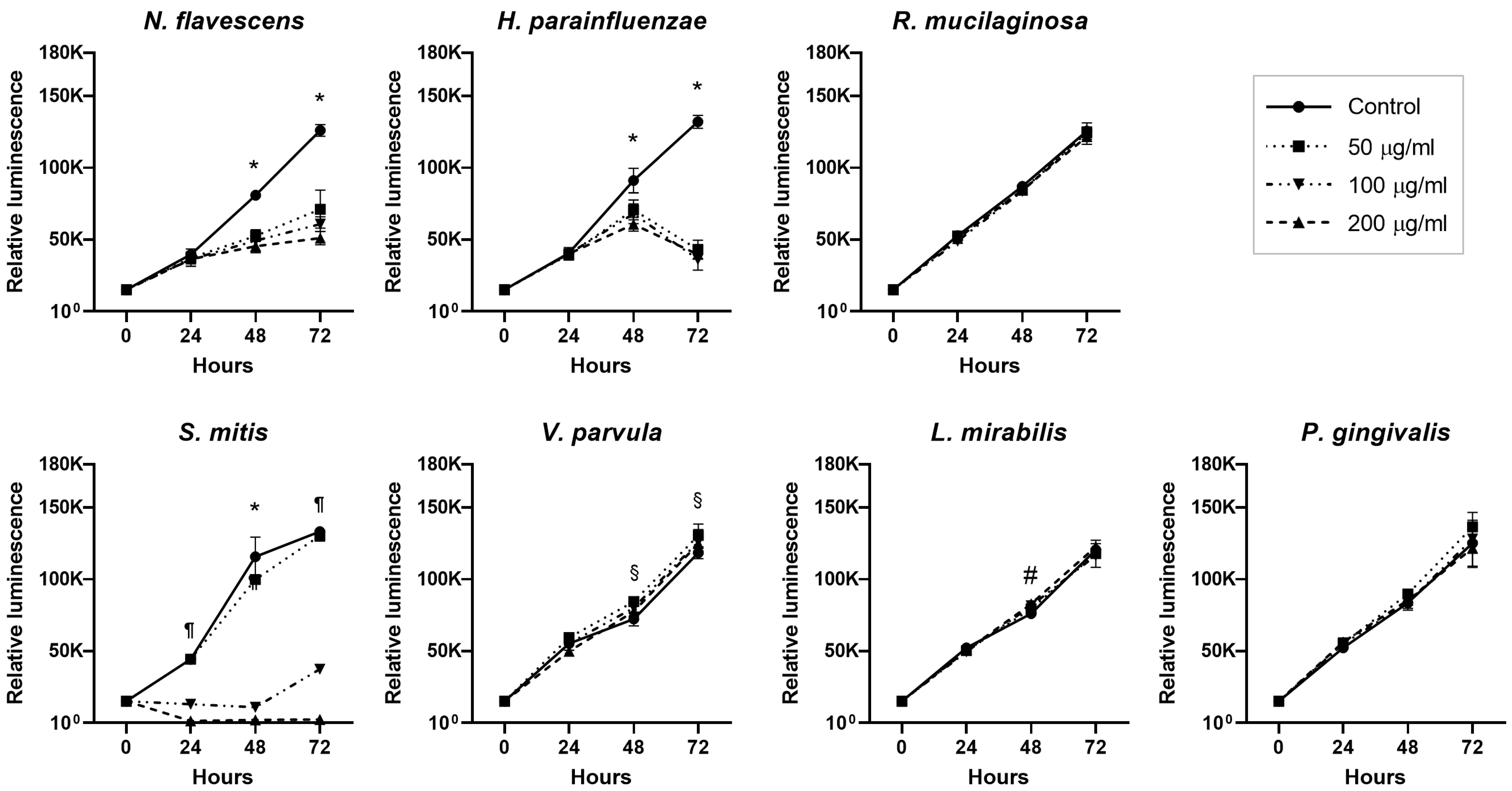

Significant difference for * all concentrations; ¶ MOI 50 and 100; # MOI 100; § MOI 10 ; ^ MOI 50; v MOI 10 and 50; $ MOI 10 and 100

Supplementary Figure 11. Effect of cocultures with bacteria on SCC25 proliferation (ATP assay)

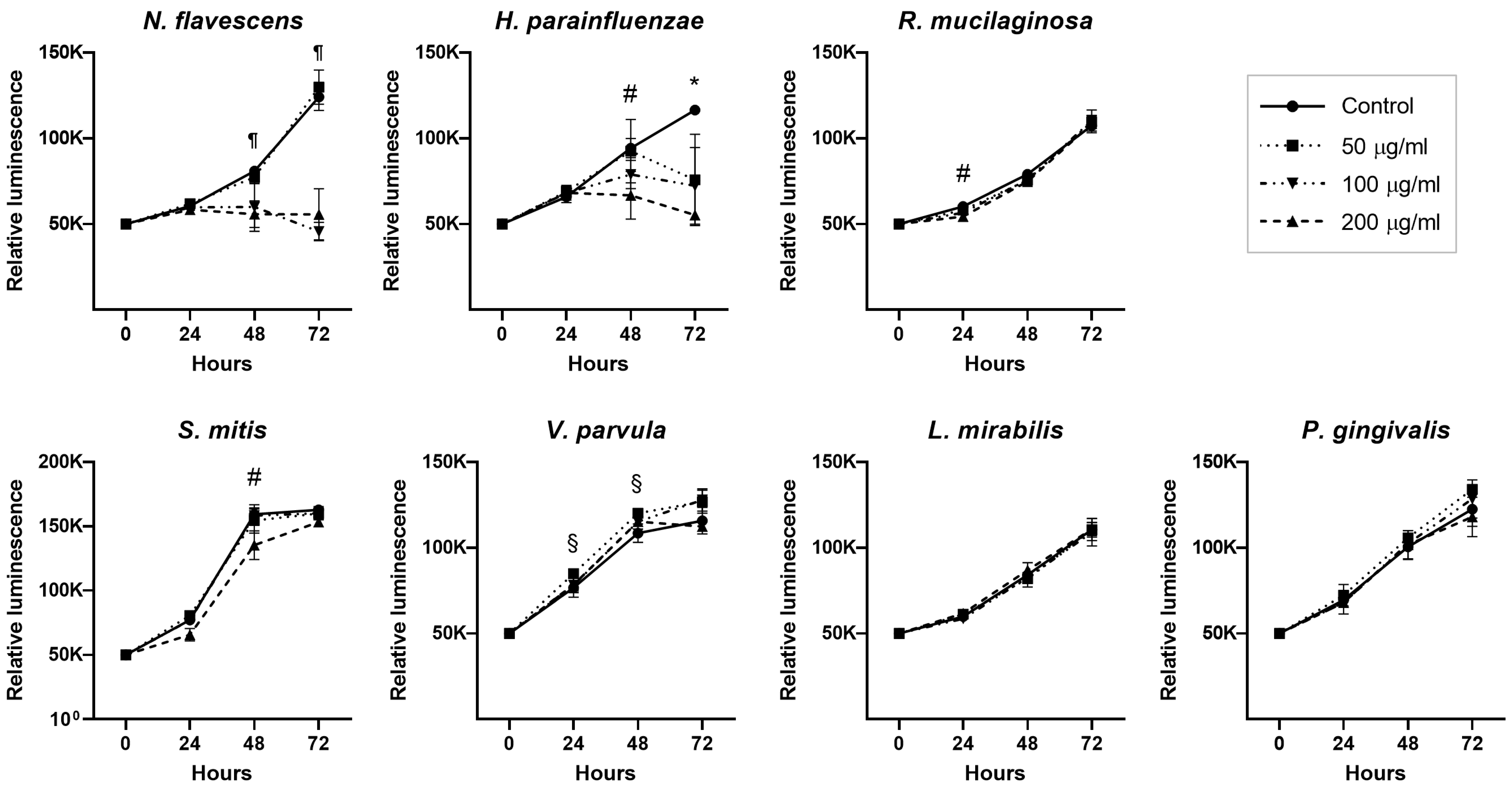

Significant difference for * all concentrations; ¶ MOI 50 and 100; # MOI 100; § MOI 10 ; ^ MOI 50; v MOI 10 and 50; $ MOI 10 and 100

Supplementary Figure 12. Effect of cocultures with bacteria on SCC4 proliferation (ATP assay)

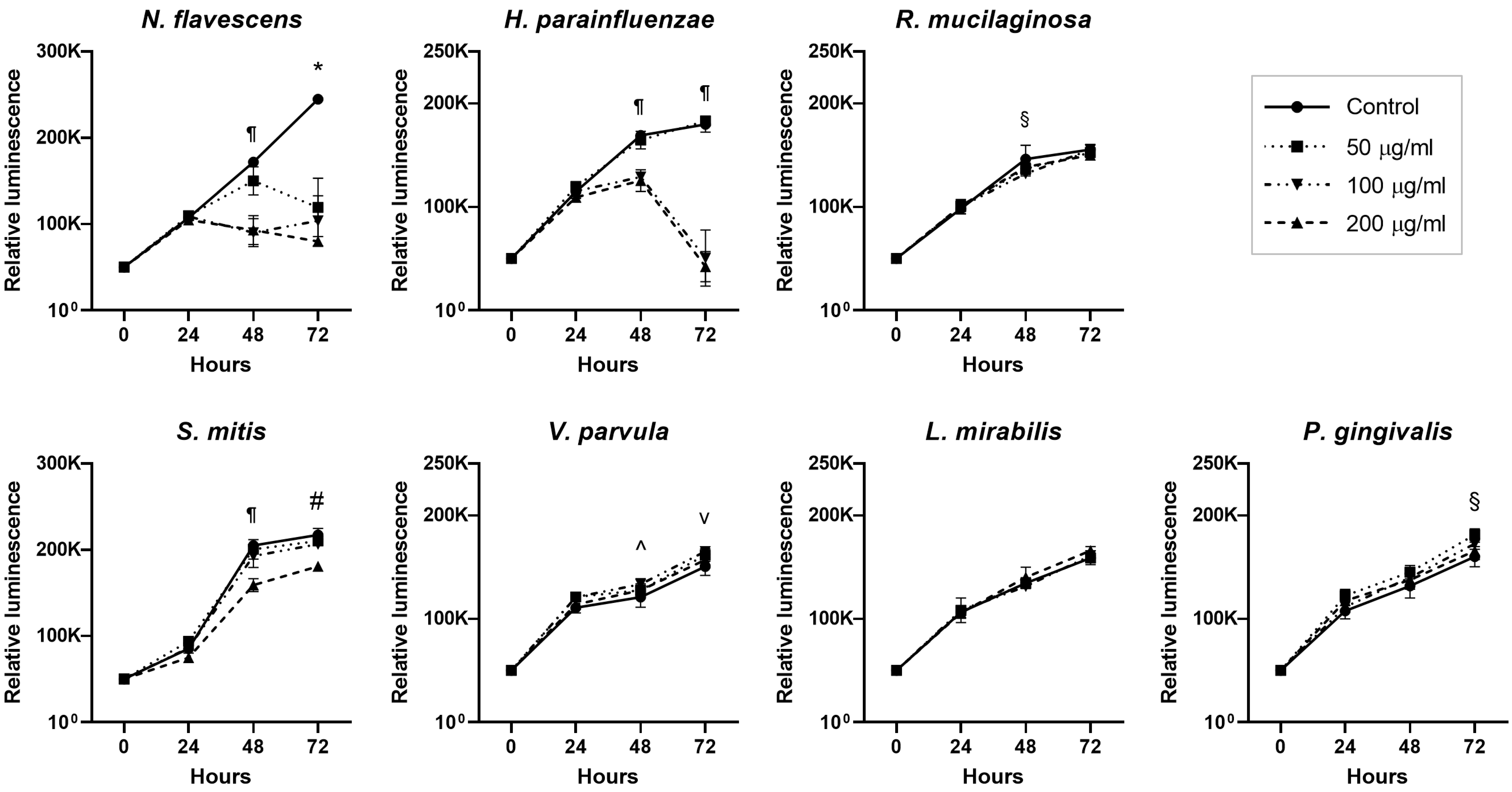

Significant difference for * all concentrations; ¶ MOI 50 and 100; # MOI 100; § MOI 10 ; ^ MOI 50; v MOI 10 and 50; $ MOI 10 and 100

Supplementary Figure 13. Effect of coculture with *S. mutans* and *S. sanguinus* on CAL27 proliferation (cell counts)

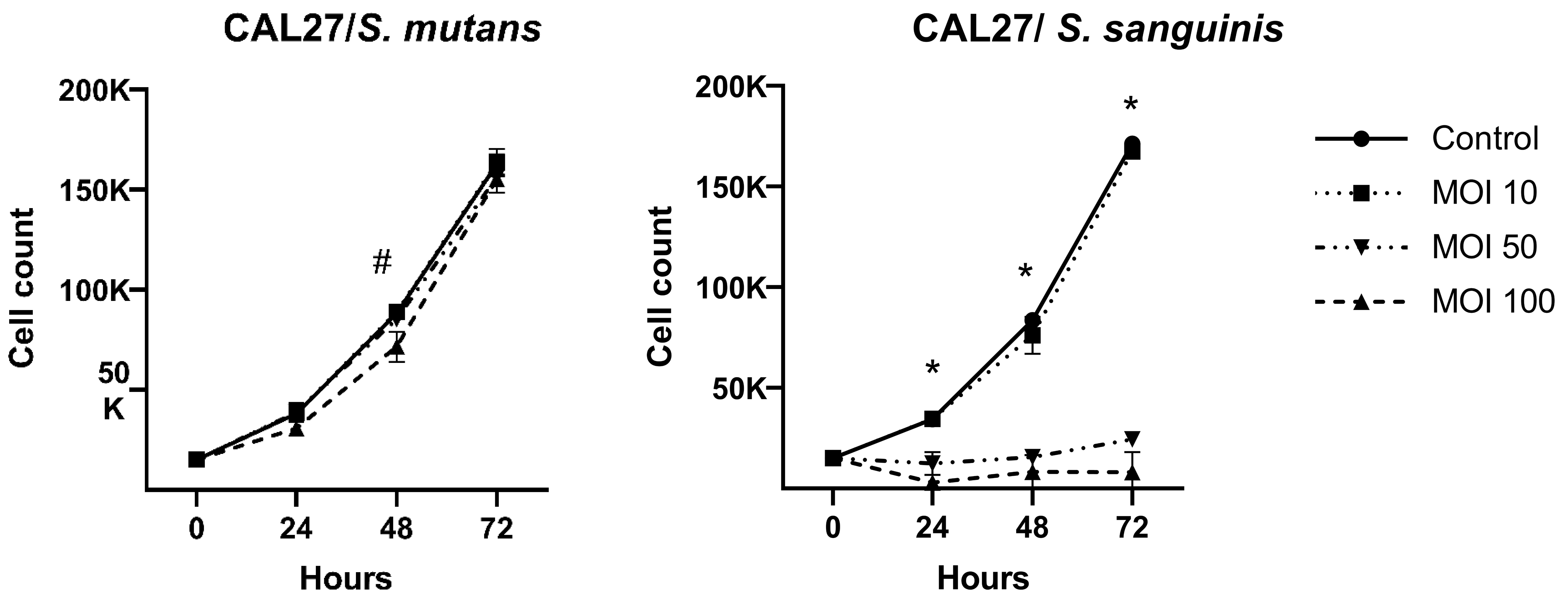

Significant difference for * all concentrations; ¶ MOI 50 and 100; # MOI 100; § MOI 10 ; ^ MOI 50; v MOI 10 and 50; $ MOI 10 and 100

Supplementary Figure 14. Percent reduction in cell proliferation by *N. flavescens* and *S. mitis* in CAL27, SCC25 SCC4 and TIGKs in cocultures.

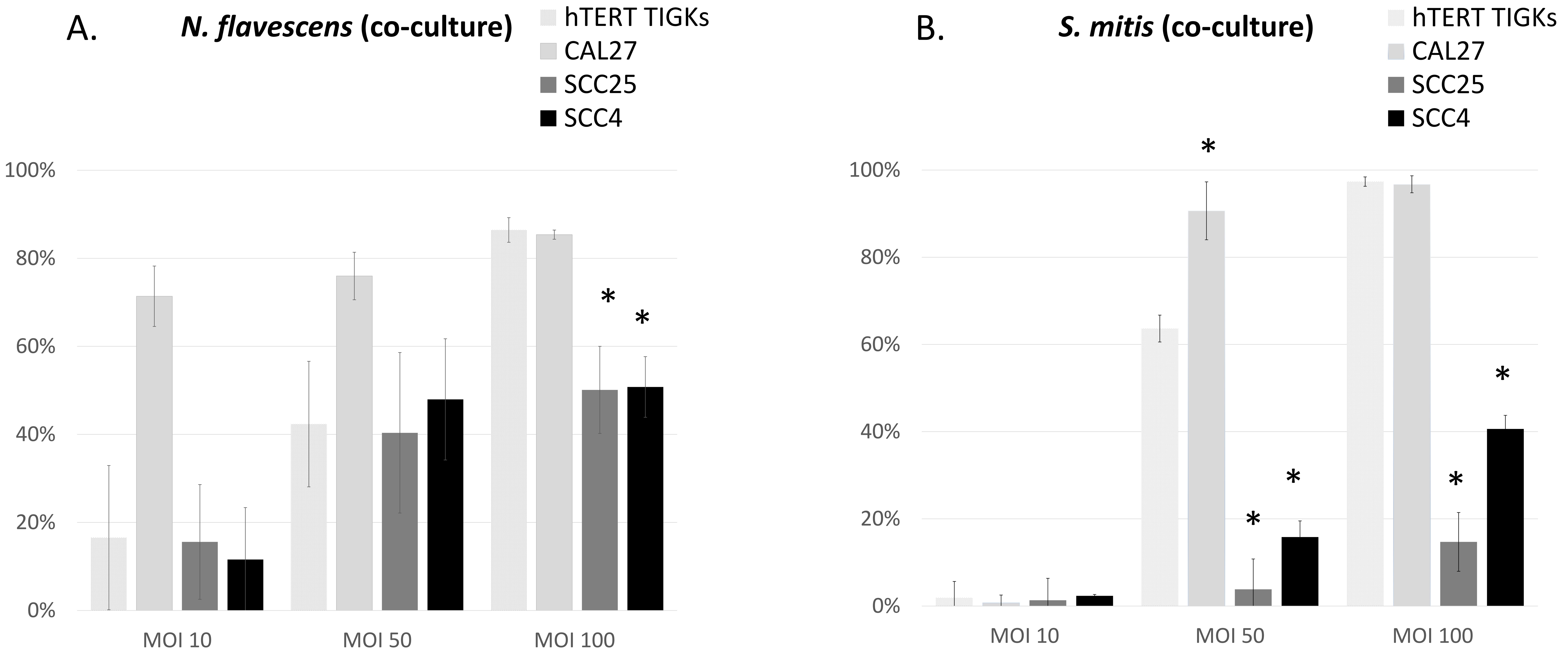

* Statistically significant ($p \leq 0.05$) compared to TIGKs; pair-wise Student's t-test.

Supplementary Figure 15. Staining of intracellular *N. flavescens* and *H. parainfluenzae* (CAL27)

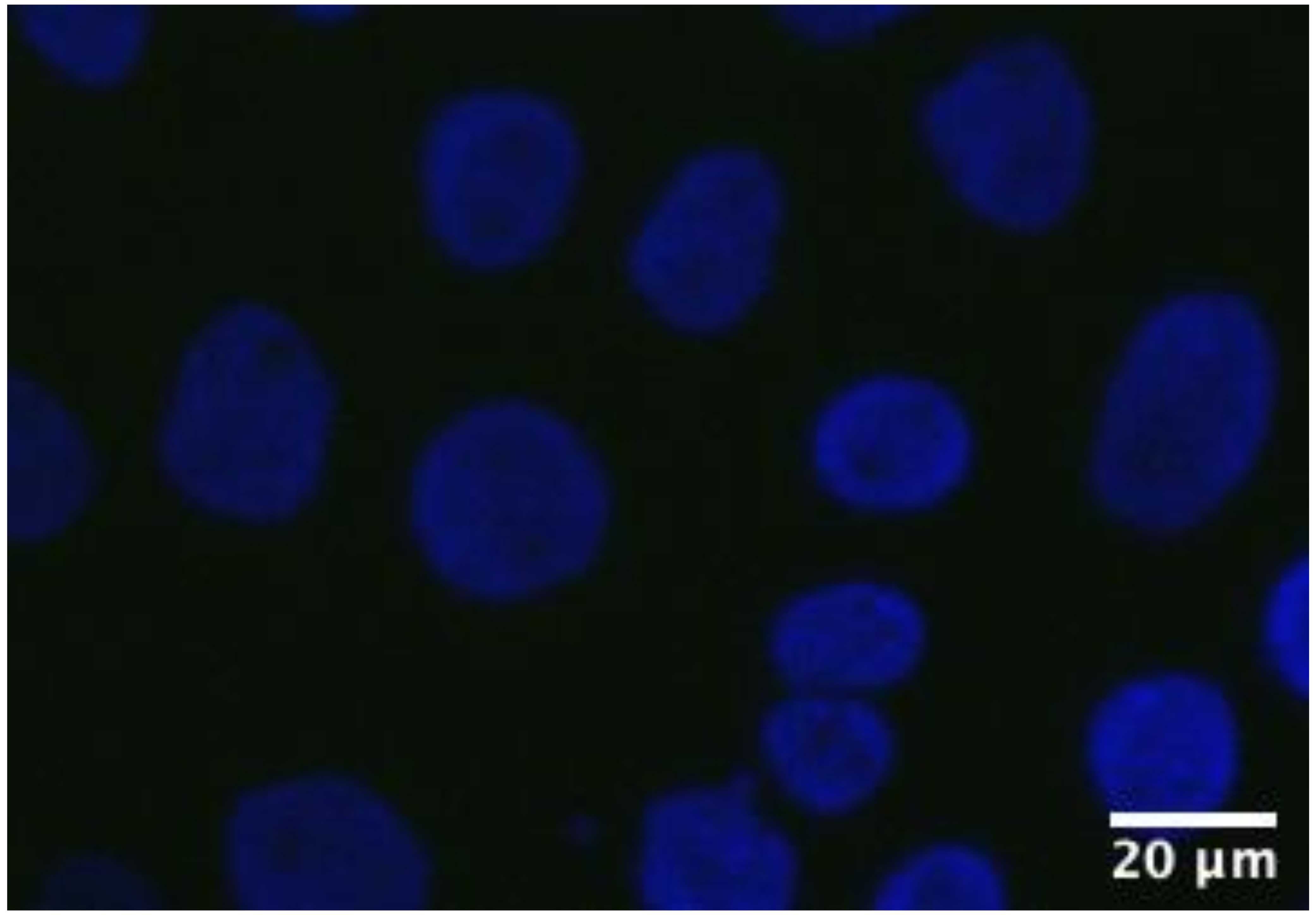

Unexposed

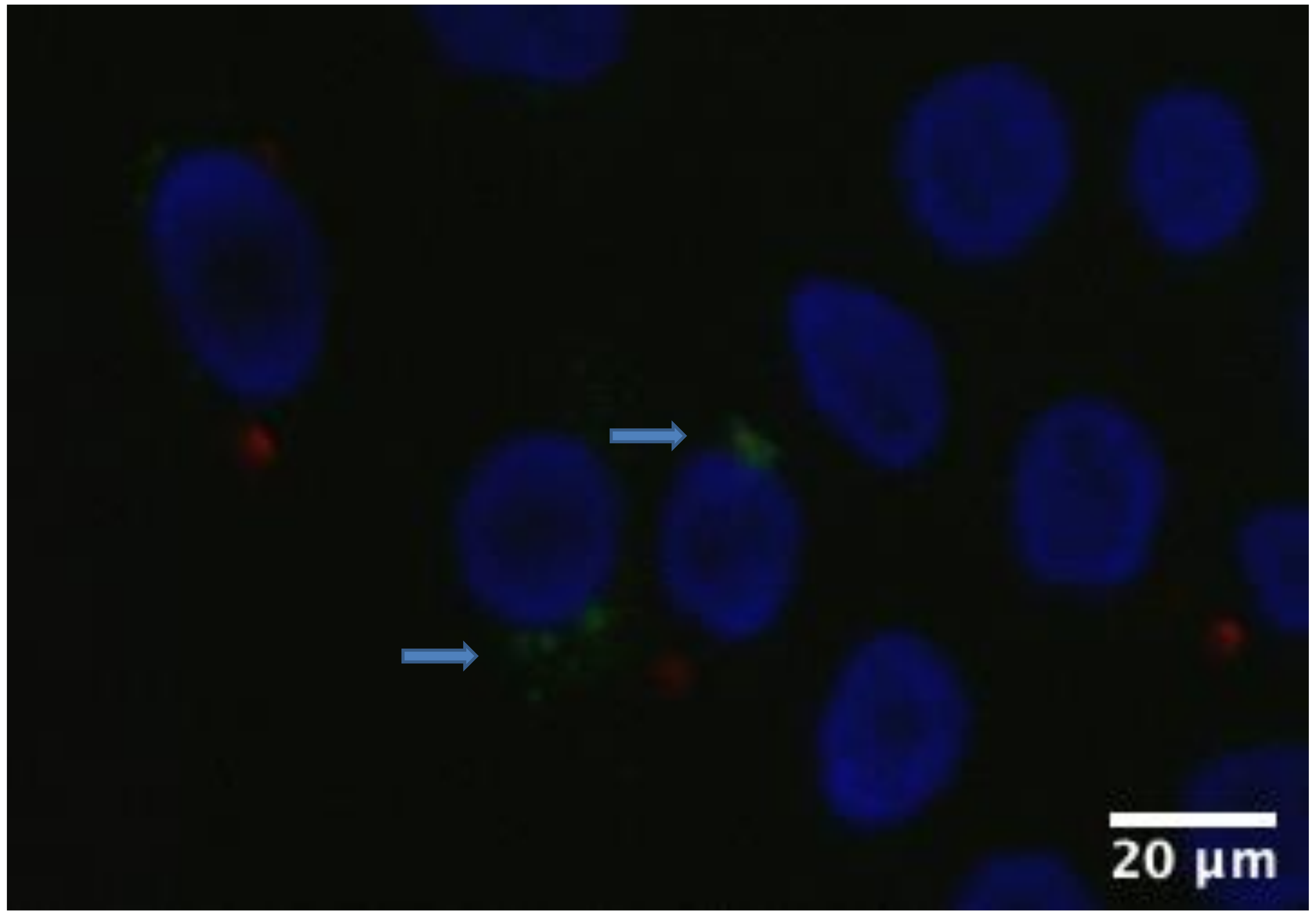

N. flavescens

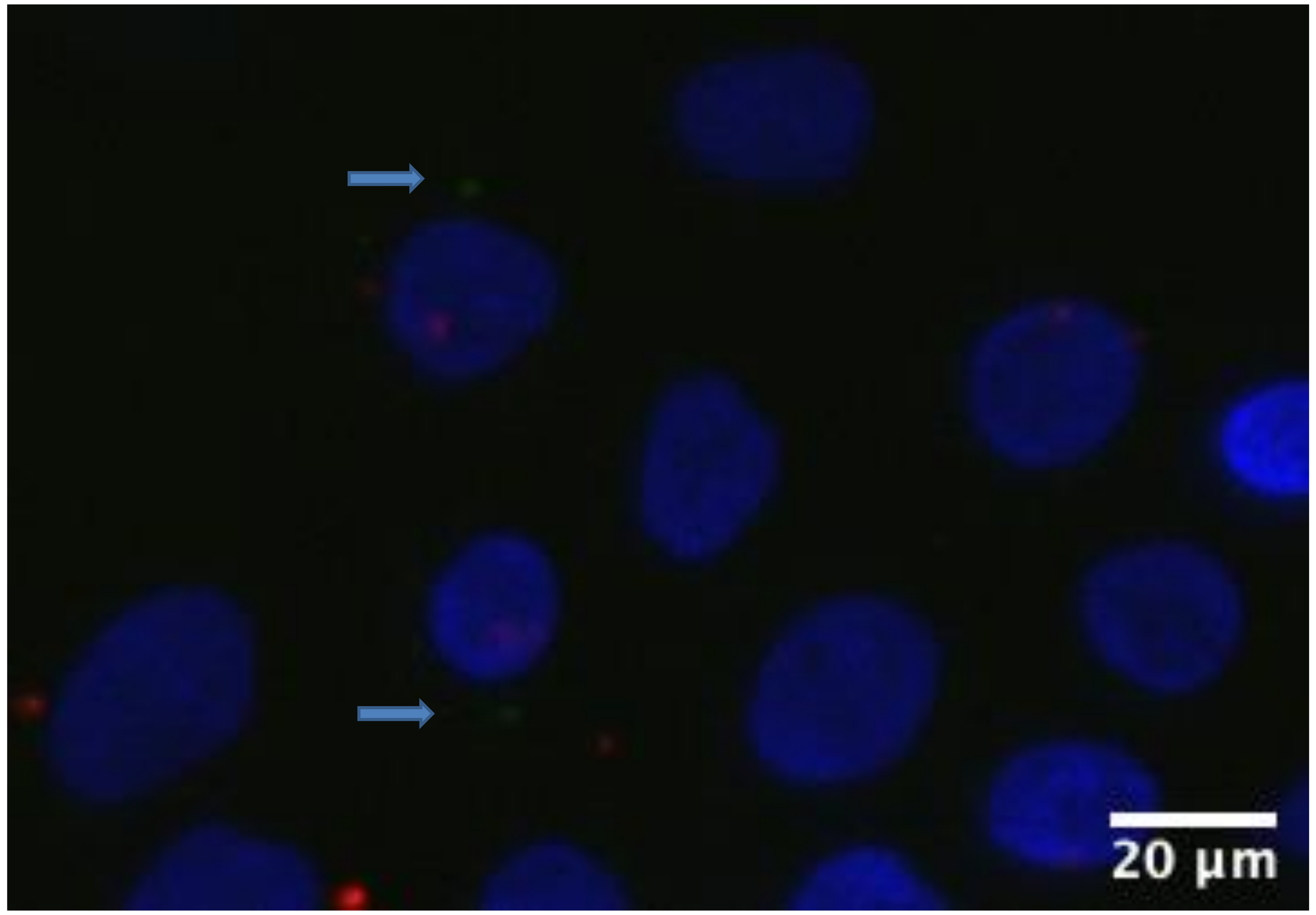

H. parainfluenzae

Color scheme
Blue : nucleus
Green : Intracellular bacteria
Red: Extracellular bacteria

Arrows in the images are pointing to intracellular bacteria with green stain

Supplementary Figure 16. Staining of intracellular *N. flavescens* and *H. parainfluenzae* (SCC25)

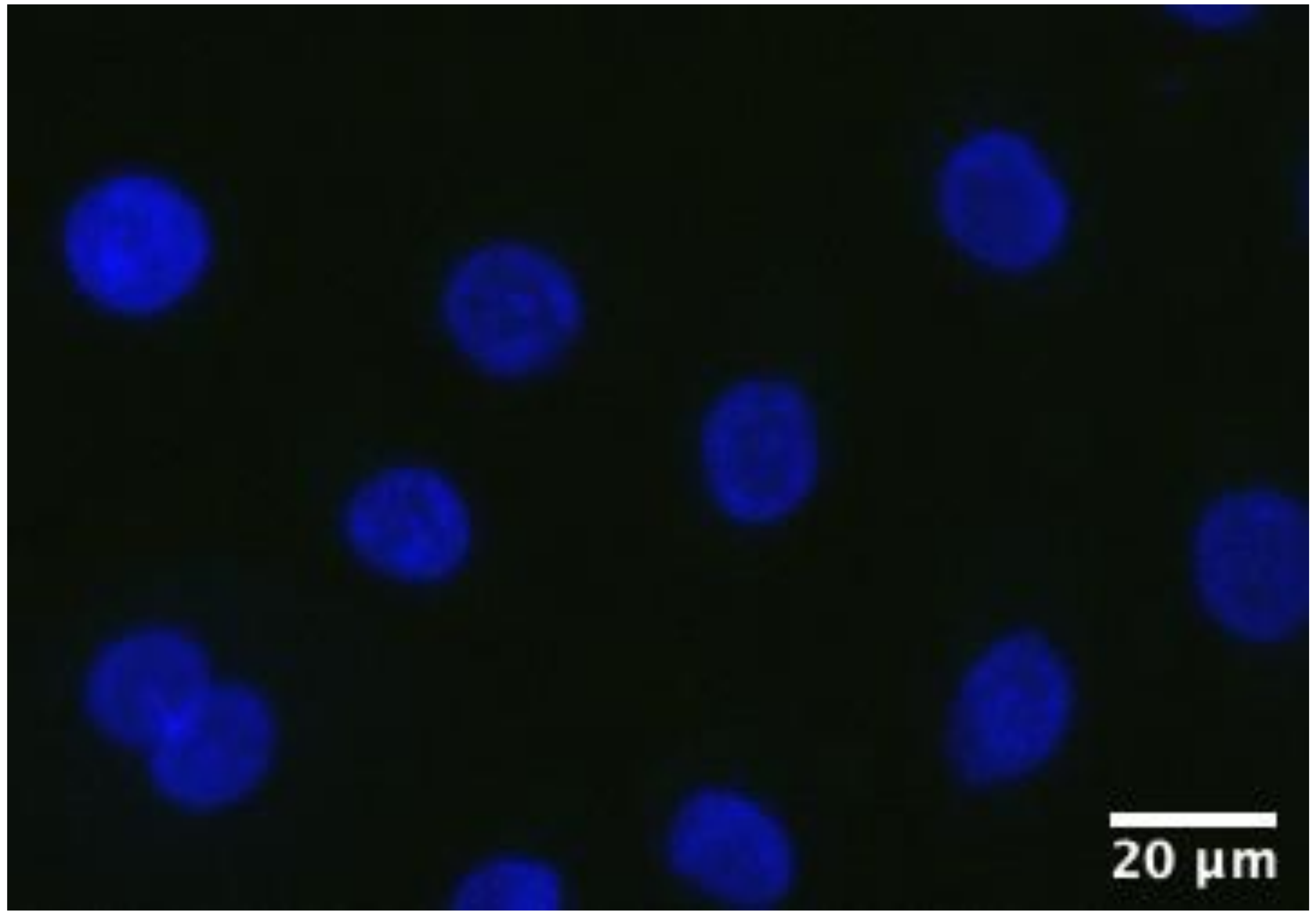

Unexposed

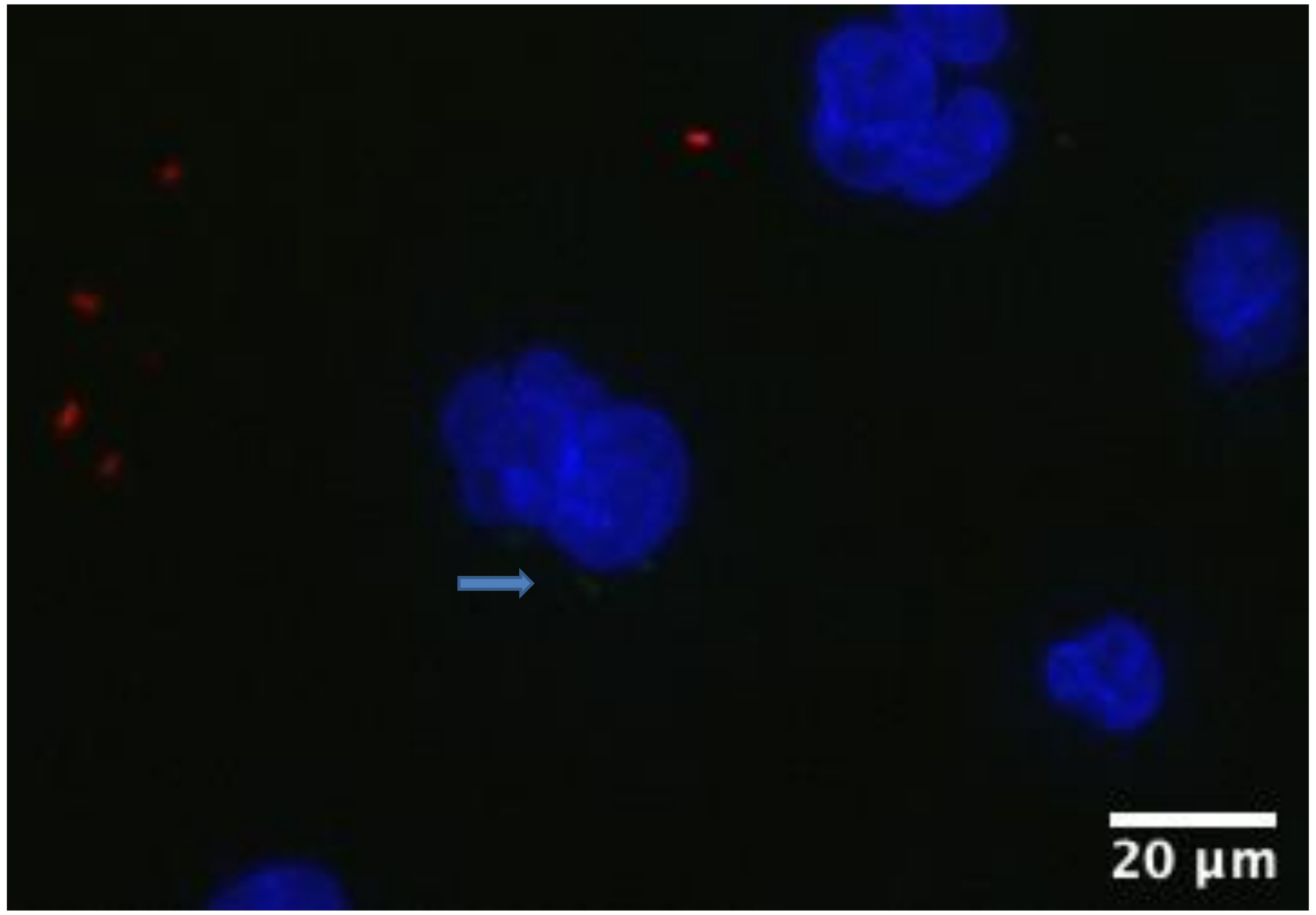

N. flavescens

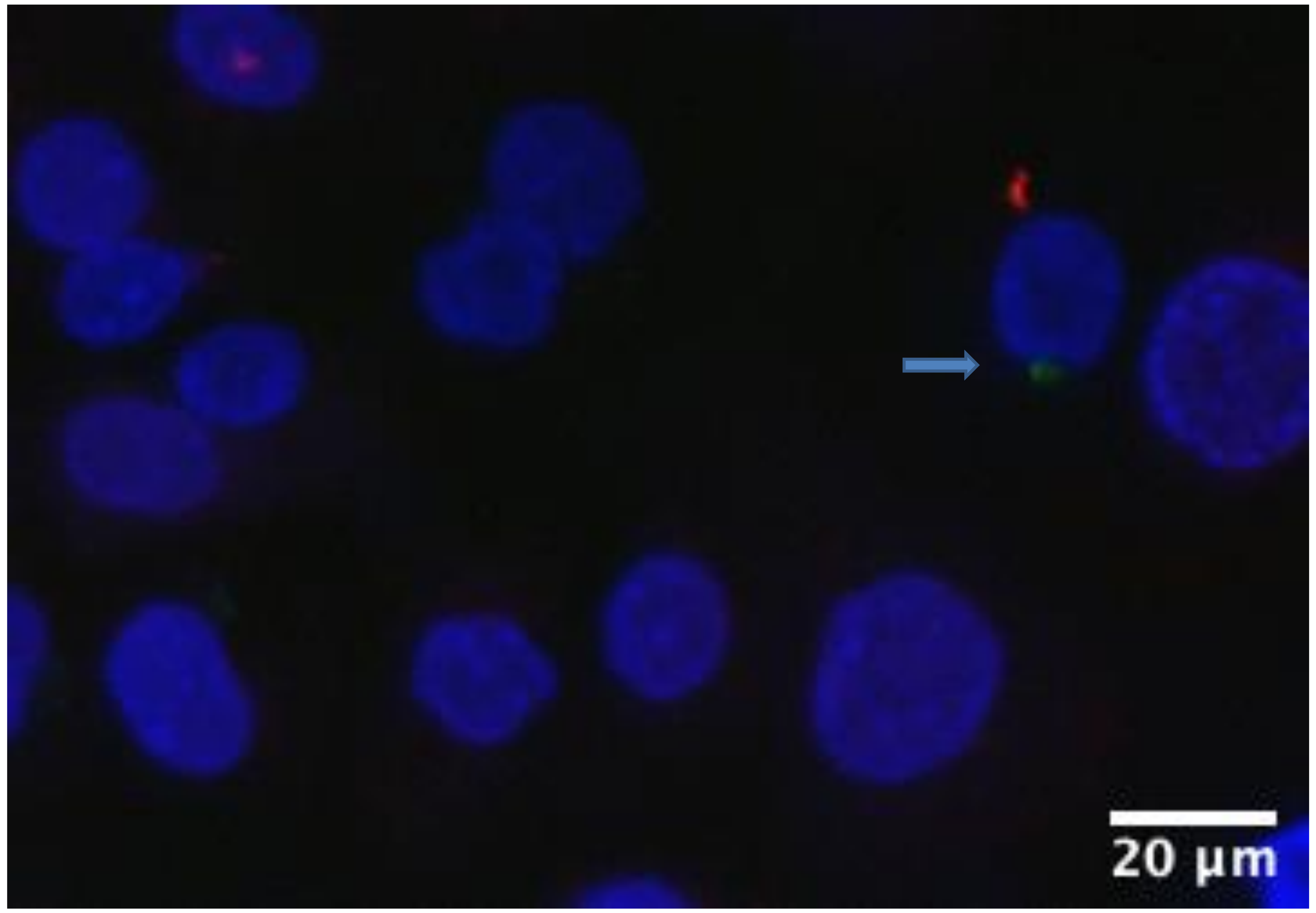

H. parainfluenzae

Color scheme
Blue : nucleus
Green : Intracellular bacteria
Red: Extracellular bacteria

Arrows in the images are pointing to intracellular bacteria with green stain

Supplementary Figure 17. Staining of intracellular *N. flavescens* and *H. parainfluenzae* (SCC4)

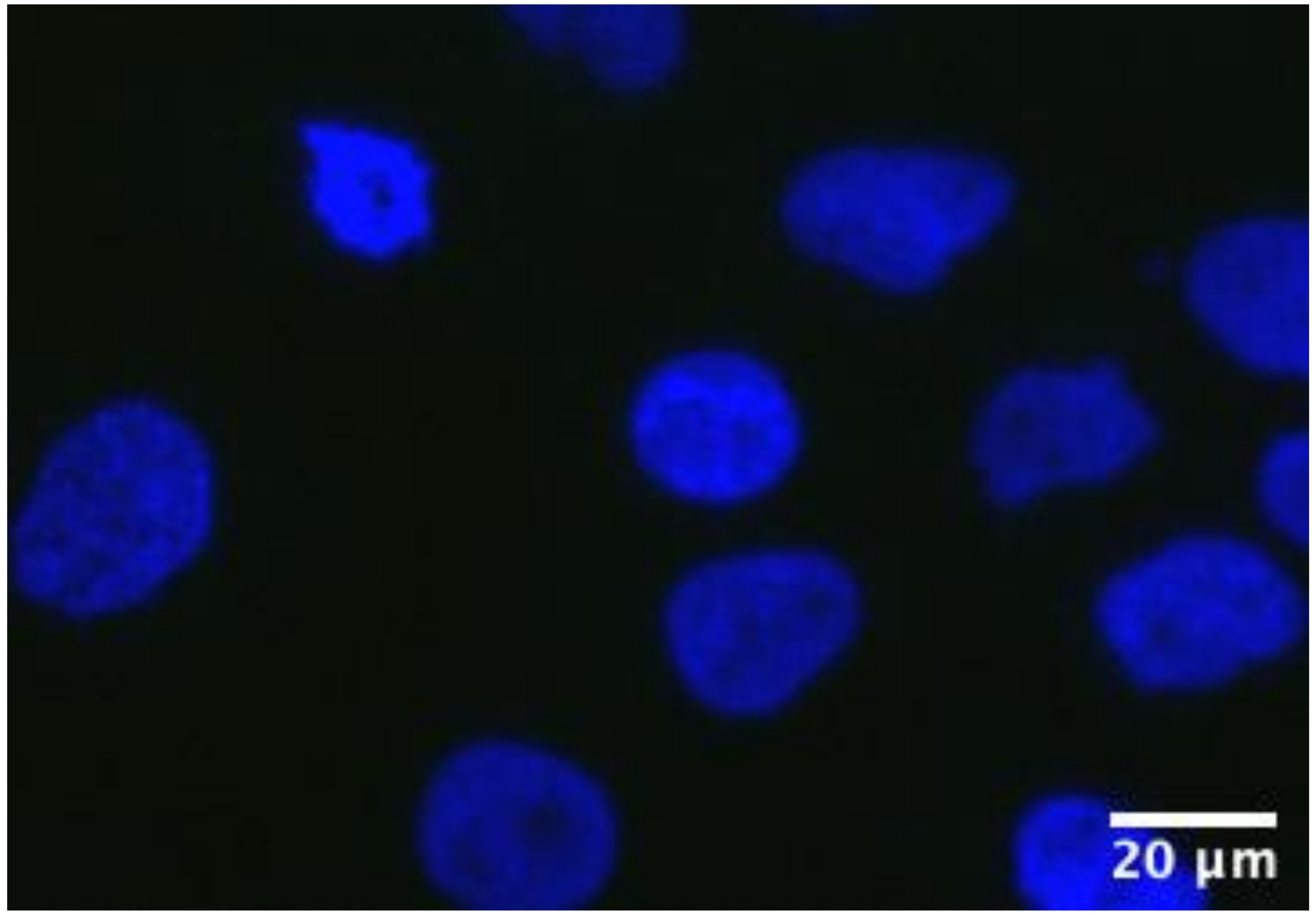

Unexposed

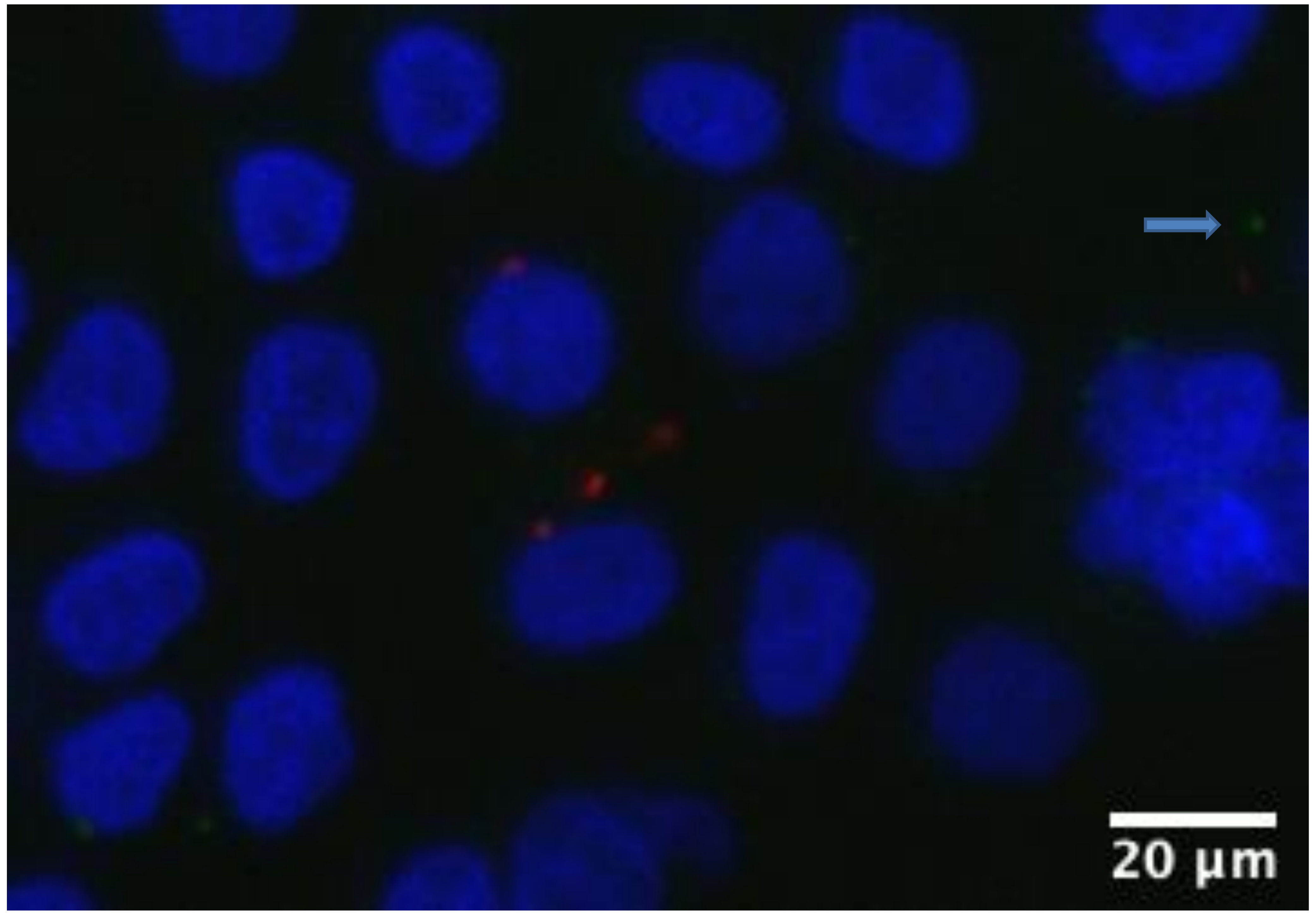

N. flavescens

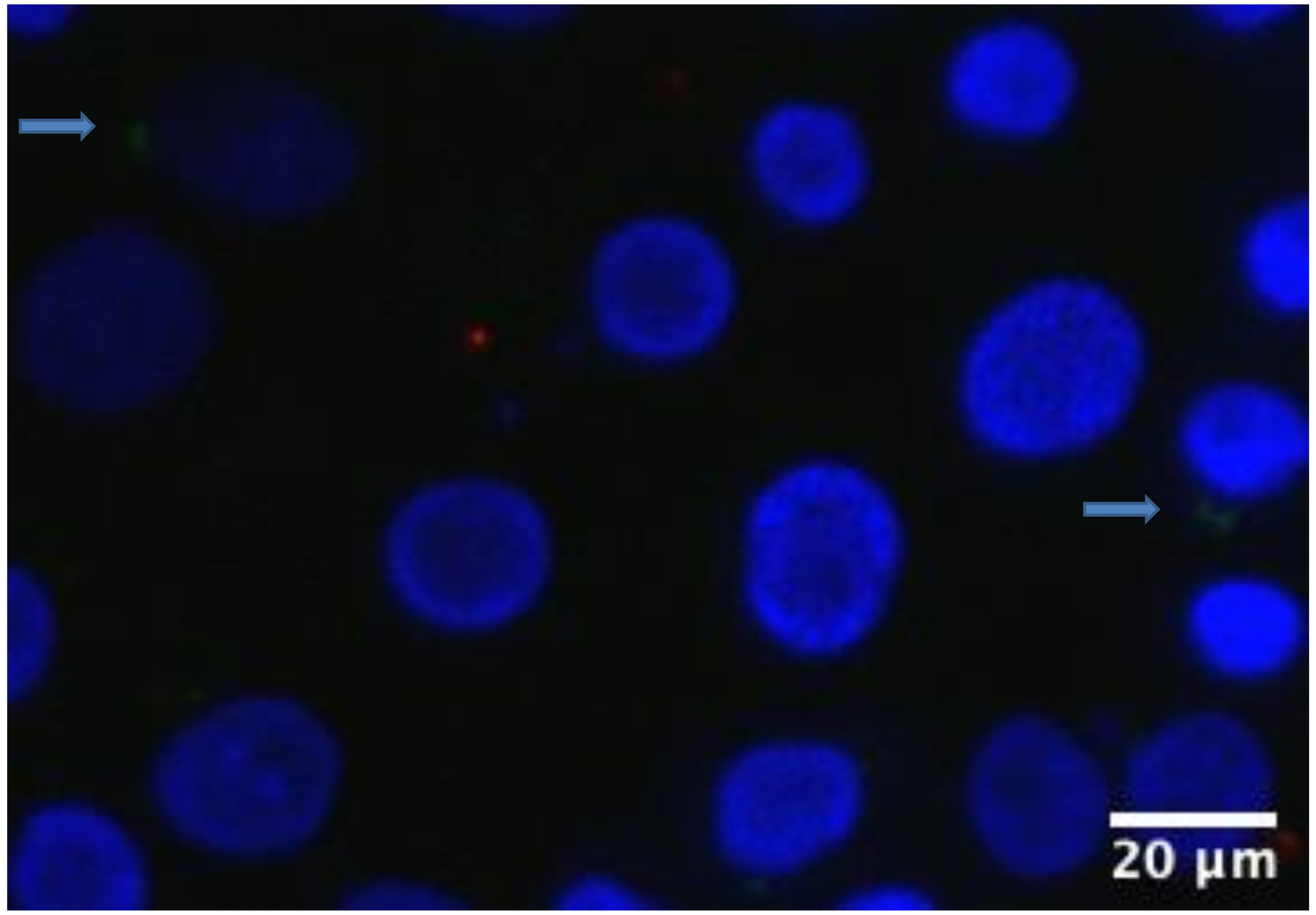

H. parainfluenzae

Color scheme
Blue : nucleus
Green : Intracellular bacteria
Red: Extracellular bacteria

Arrows in the images are pointing to intracellular bacteria with green stain

Supplementary Figure 18. Effect of bacterial lysates on gene expression in CAL27

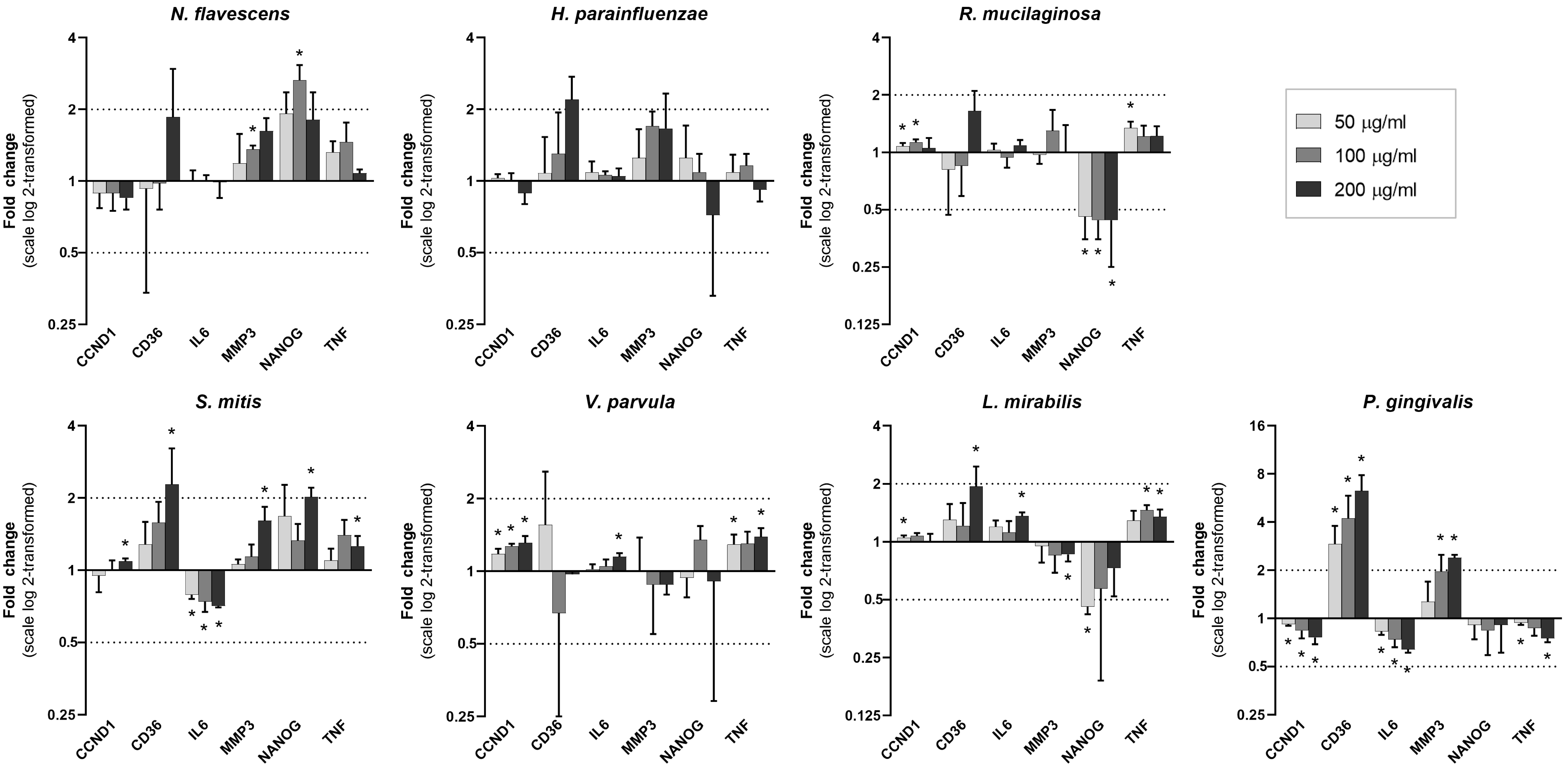

* Significant difference relative to control (P< 0.05)

Supplementary Figure 19. Effect of bacterial lysates on gene expression in SCC25

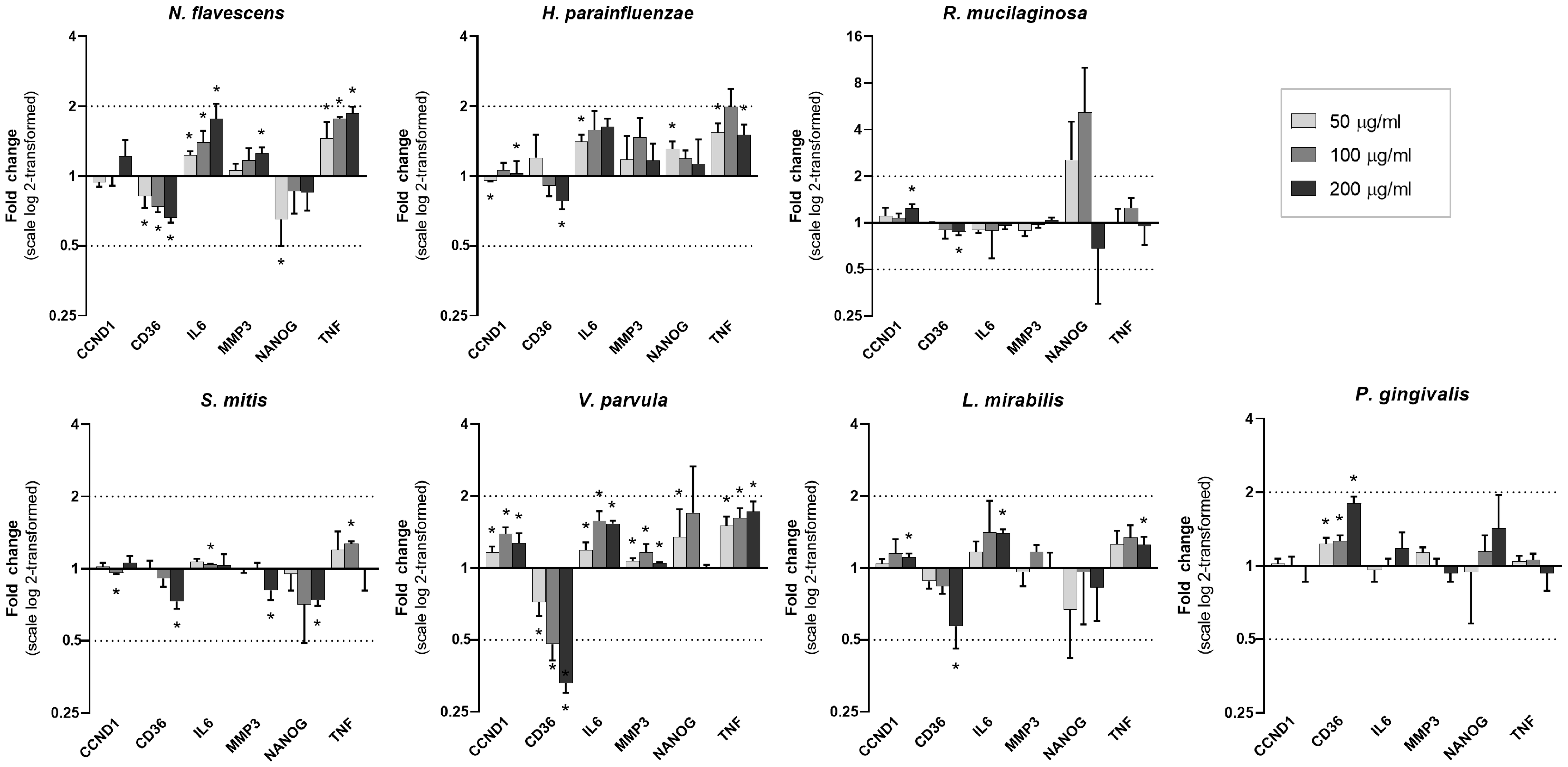

* Significant difference relative to control (P< 0.05)

Supplementary Figure 20. Effect of bacterial lysates on gene expression in SCC4

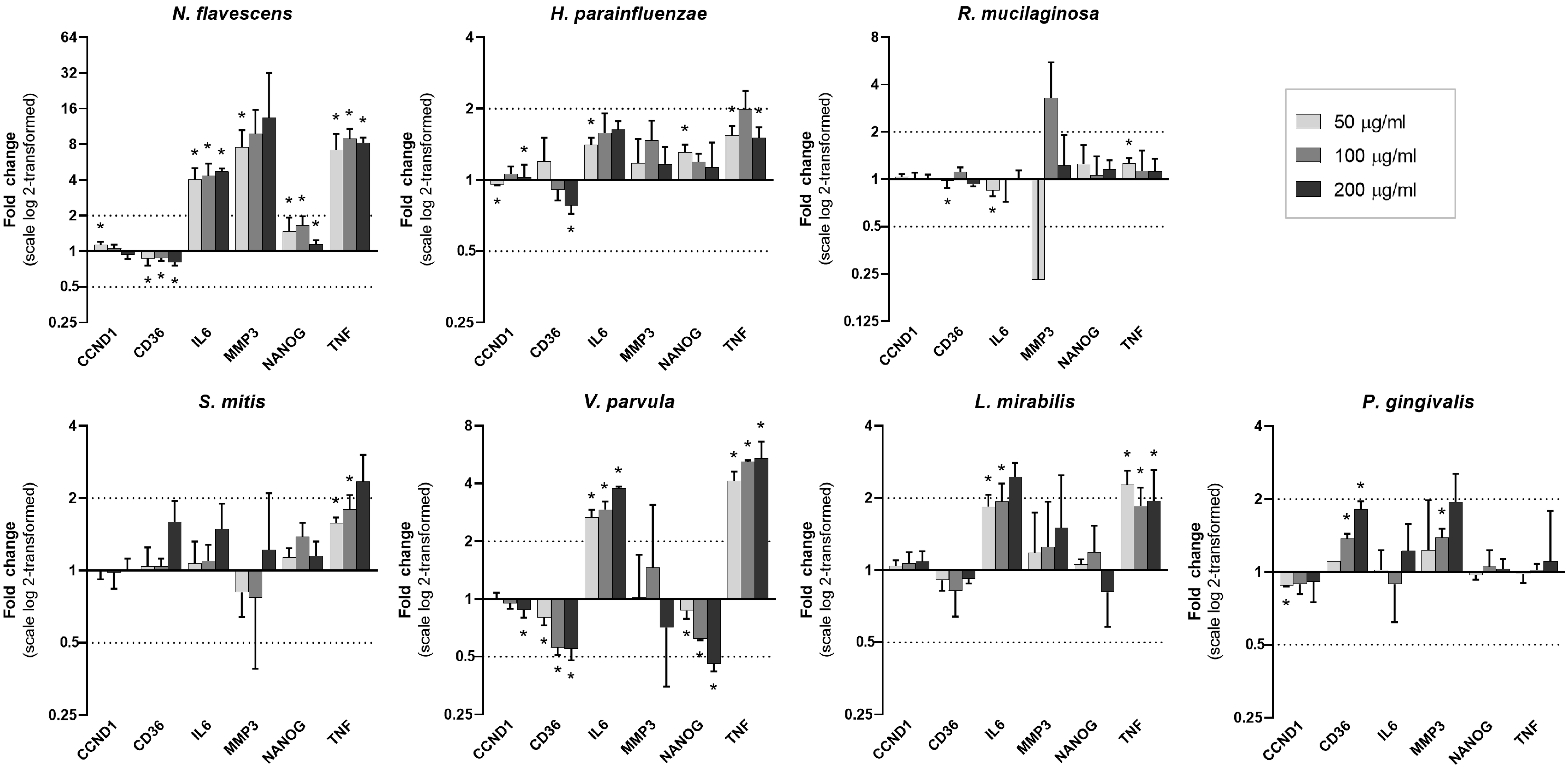

* Significant difference relative to control (P< 0.05)

Supplementary Figure 21. Effect of cocultures with bacteria on gene expression in CAL27

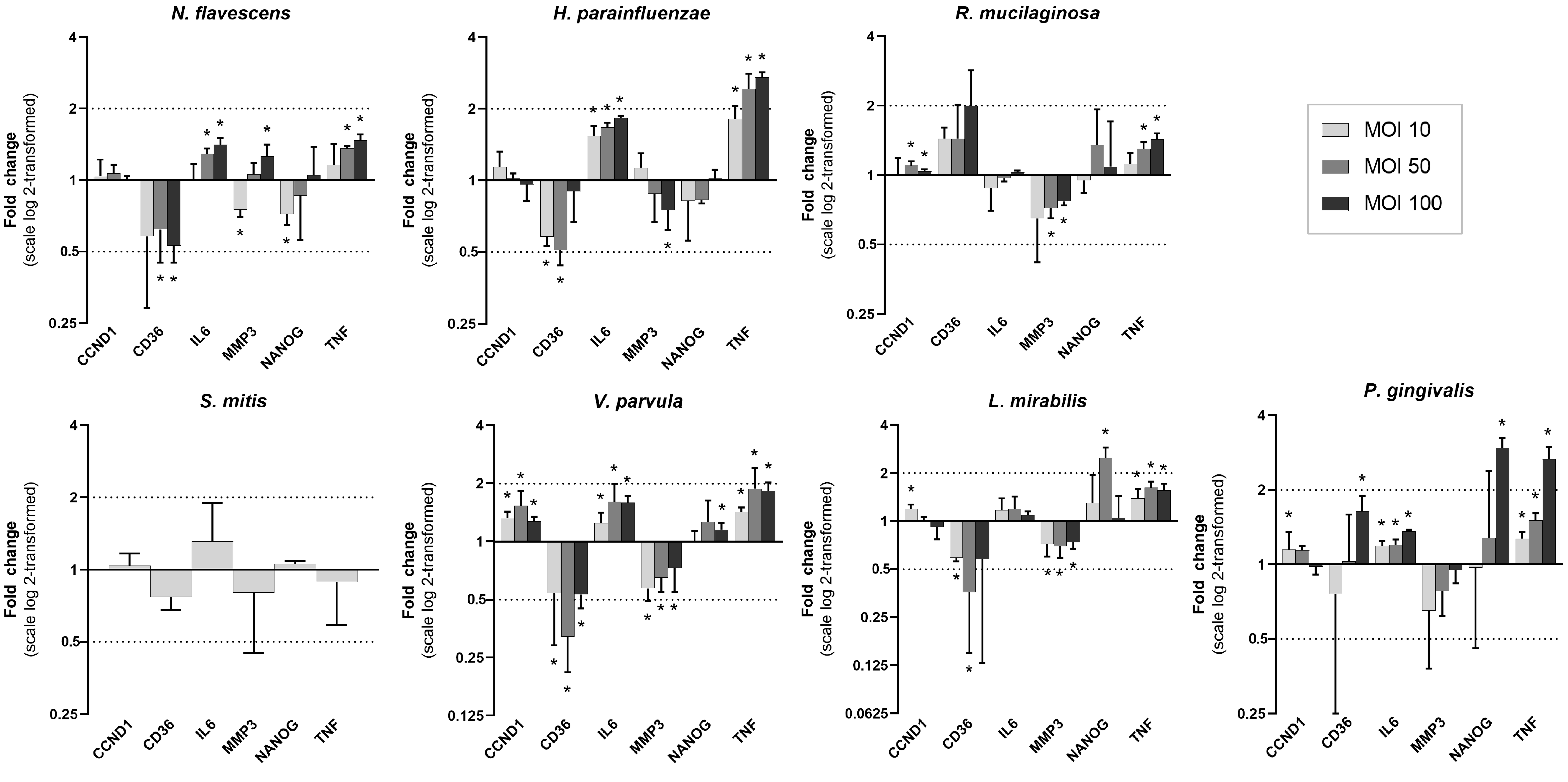

* Significant difference relative to control (P< 0.05)

Supplementary Figure 22. Effect of cocultures with bacteria on gene expression in SCC25

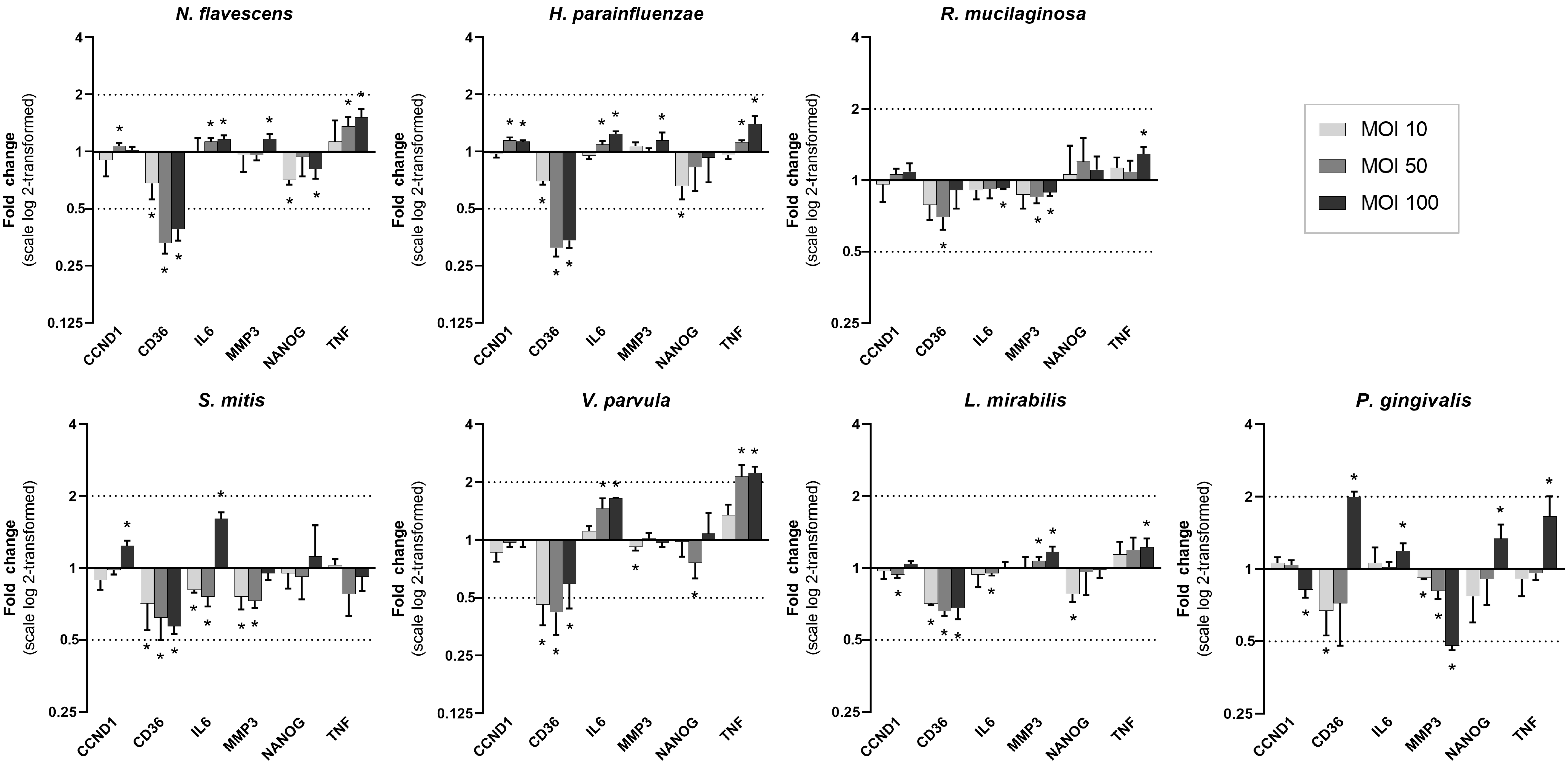

* Significant difference relative to control (P< 0.05)

Supplementary Figure 23. Effect of cocultures with bacteria on gene expression in SCC4

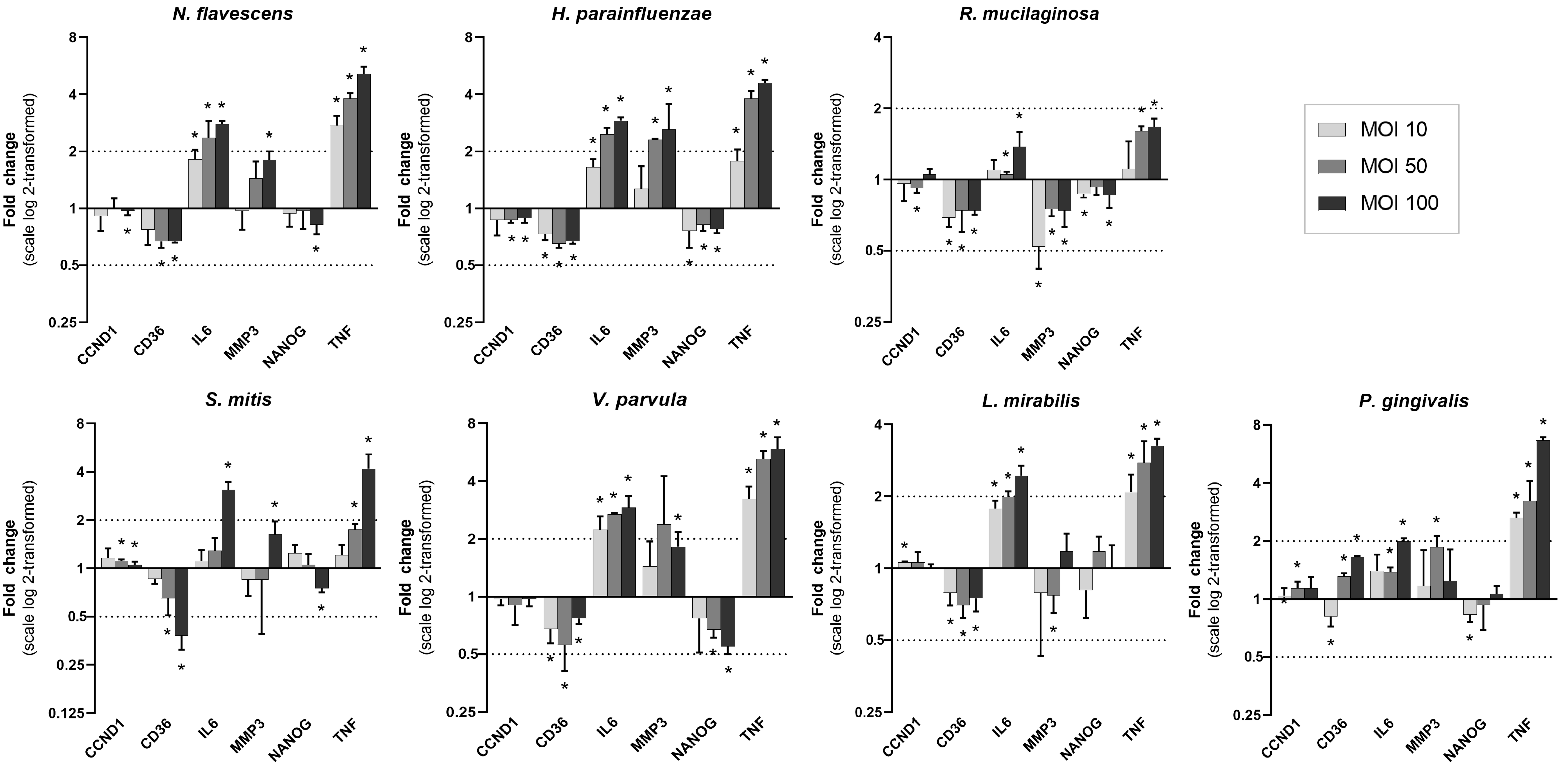

* Significant difference relative to control (P< 0.05)

Supplementary Figure 24. Effect of exposure to bacteria at MOI 100 on IL6 and TNFα concentrations in culture supernatant

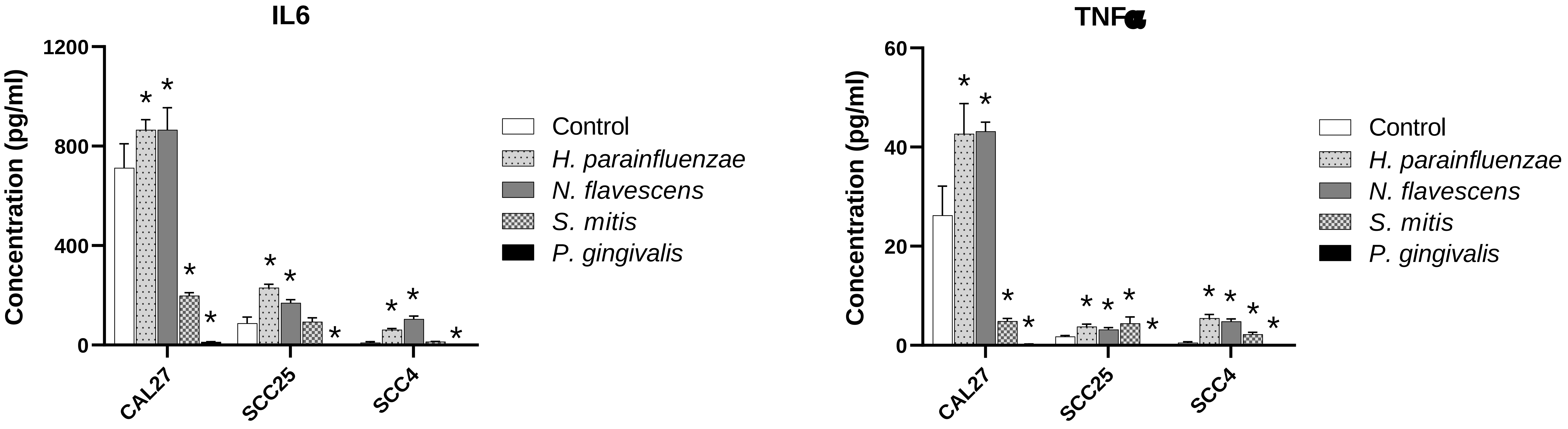

* Significant difference relative to control (P< 0.05)
