# Supplementary material for: Screening of Health-Associated Oral Bacteria for Anticancer Properties in vitro
Source: Front Cell Infect Microbiol. 2020 Oct 6;10:575656. doi: 10.3389/fcimb.2020.575656 (PMC7573156; doi:10.3389/fcimb.2020.575656)
Supplement: Supplementary file 2 [file Data_Sheet_1.docx]

**Supplementary methods 1. Preparation of lysates**

Following method was used for culture and preparation of lysates from capnophilic bacteria ***Rothia mucilaginosa****,* ***Streptococcus mitis, Neisseria flavescens*** and ***Hemophilus parainfluenzae*** individually

1. Pure culture of bacteria was grown overnight in BHI media supplemented with 0.5 % Hemin, 0.1% Vitamin K and 1% Isovitalex (BHKI) media at 37 ̊C in 5% CO_2_ with shaking.
2. After overnight growth, culture was diluted in fresh BHKI culture media till the optical density at 600nm (OD_600nm_) was 0.1 in about 30-50 ml of total volume and incubated till OD_600nm_ was between 0.6- 0.7.
3. As soon as culture reached required OD_600nm_, about 100µl of it was spread on a BHI agar plate supplement with Hemin, Vitamin K and Isovitalex and was incubated at 37 ̊C and 5% CO_2_ to check for contamination, if any.
4. Remaining sample was centrifuged immediately at 5000 rpm for 12 minutes.
5. After discarding the supernatant, the pellet was suspended in Phosphate Buffered Saline (PBS) and centrifugated at 5000 rpm for 5 minutes at 4°C. This process was repeated two more times.
6. After discarding the supernatant from third washing pellet was resuspend in 1 ml of 15 mM HEPES (4-(2-hydroxyethyl)-1-piperazineethanesulfonic acid) buffer and transferred to tubes containing sterile 100-micron zirconium beads ([PFAW 100-100-02U], OPS diagnostics).
7. Samples were homogenized in a bead beater (FastPrep-24^TM^, MP Biomedicals) at a speed of 6.0 m/s for 15 seconds and cooled on ice for 1 minute, it was repeated 3 times for gram negative bacteria and 6 times for gram positive bacteria.
8. Homogenates were filtered through low protein binding 0.2μ PES syringe filters, protein concentration was determined by measuring absorbance at a wavelength of 280 nm on a Nanodrop (ThermoFisher Scientific, USA) and lysates were immediately stored at -80°C till further use.

For ***Lautropia mirabilis*** pure culture was grown on BHKI agar plat at 37°C in aerobic conditions for 24 hours. Later a few colonies were suspended in BHKI media using a polystyrene swab by crushing them against the walls of the tube to disperse the bacteria well in solution and incubated for 14-16 hours at 37°C in aerobic conditions with shaking, till OD_600nm_ was between 0.35-0.4. and steps 4-10 of from preparation of lysates from capnophilic bacteria were followed.

For ***Porphyromonas gingivalis*** culture was grown on BHKI agar plate for 2-3 days at 37 ̊C in anaerobic conditions till appearance of colonies. A few colonies were suspended in BHKI media, grown at 37 ̊C in anaerobic conditions overnight and culture was diluted in fresh BHKI media to OD_600nm_ of 0.1 and grown again in same conditions till OD_600nm_ reached between 0.6-0.7 and steps 4-10 mentioned in preparation of lysates from capnophilic bacteria followed.

***Veillonella parvula*** was grown on Brain Heart Infusion supplemented with 1.5% of 60% sodium lactate (BHI-NaL) agar in anaerobic conditions at 37°C for 24-48 hours till the appearance of colonies. All the colonies were collected and suspended in liquid BHI-NaL media using sterile polystyrene swab and steps 4-10 mentioned in preparation of lysates from capnophilic bacteria were followed.

**Supplementary methods 2. Growth of bacteria for infection**

1. **Capnophilic bacteria**: Pure cultures of ***R. mucilaginosa****,* ***S. mitis****,* ***N. flavescens*** and ***H. parainfluenzae*** were grown overnight in BHI media supplemented with 0.5 % Hemin, 0.1% Vitamin K and 1% Isovitalex (BHKI) media at 37 ̊C in presence of 5% CO_2_ with shaking. Each culture was diluted to OD_600nm_ of 0.1 in fresh BHKI media and allowed to grow for another 2-4 hours till OD_600nm_ of 0.3-0.4 was achieved. At this stage the bacteria were centrifuged at 5000 rpm for 10 minutes and were suspended in Dulbecco’s modified Eagle’s medium (DMEM) supplemented with 10% Fetal Bovine Serum (FBS) and 2.5 mM L-Glutamine. OD_600nm_ was measured and cultures were diluted if required and were added to experiments to achieve required modalities of infection (MOI) for gene expression and proliferation experiments.
2. **Anaerobic bacteria: *P. gingivalis*** was grown on BHKI agar plate for 2-3 days till the appearance of colonies at 37 ̊C in anaerobic conditions. A few colonies were suspended in BHKI media, grown at 37 ̊C in anaerobic conditions overnight and culture was diluted in fresh BHKI media to get an OD_600nm_ of 0.1 and grown again in same conditions till OD_600nm_ was between 0.3-0.4. At this stage bacterial cells were pelleted by centrifuging at 5000 rpm for 10 minutes and were suspended homogenously in DMEM media supplemented with 10% Fetal Bovine Serum (FBS) and 2.5 mM L-Glutamine. OD_600nm_ was measured and cultures were diluted if required and were added to experiments to achieve required modalities of infection (MOI) for gene expression and proliferation experiments.

***V. parvula*** was grown on BHI-NaL agar in anaerobic conditions at 37°C for 24-48 hours till the appearance of colonies. All the colonies were collected and suspended in liquid BHI-NaL media using sterile polystyrene swab and grown at 37 ̊C in anaerobic conditions overnight. In morning bacterial cells were pelleted down by centrifuging at 5000 rpm for 10 minutes and were processed as mentioned for *P. gingivalis* after preparing homogenous suspension in DMEM media supplemented with 10% Fetal Bovine Serum (FBS) and 2.5 mM L-Glutamine.

1. **Aerobic bacteria:** Pure culture of ***L. mirabilis*** was grown on BHKI agar plate at 37°C in aerobic conditions for 16-20 hours. A few colonies were suspended in DMEM media supplemented with 10% Fetal Bovine Serum (FBS) and 2.5 mM L-Glutamine media using a polystyrene swab by crushing them against the walls of the tube to disperse the bacteria well in solution, OD_600nm_ was measured and suspension was diluted if required and was added to experiments to achieve required modalities of infection (MOI)for gene expression and proliferation experiments.

**Supplementary methods 3. Antibiotics concentrations used for bacterial strains**

For determining the sub – minimum inhibitory concentration (sub-MIC), each bacterium was grown in a range of different Penicillin/ Streptomycin concentrations for 24 and 48 hours and the growth was recorded by measuring OD_600nm_. The antibiotic concentration which was just lower than the MIC was selected as sub-MIC for that bacterium. Below are the tables showing sub_MIC of antibiotics for A. Proliferation assay and B. Gene expression assay.

1. Sub-MICs for proliferation and ATP assays

| **Species** | **Penicillin/Streptomycin concentration (IU/ml)** |
| --- | --- |
| *H. parainfluenzae* | 1.5625 |
| *N. flavescens* | 1.5625 |
| *L. mirabilis* | 0.78125 |
| *R. mucilaginosa* | 0.78125 |
| *V. parvula* | 0 |
| *P. gingivalis* | 0 |
| *S. mitis* | 0.2 |

1. Sub-MICs for gene expression assays

| **Species** | **Penicillin/Streptomycin concentration (IU/ml)** |
| --- | --- |
| *H. parainfluenzae* | 3.125 |
| *N. flavescens* | 3.125 |
| *L. mirabilis* | 1.5625 |
| *R. mucilaginosa* | 0.78125 |
| *V. parvula* | 0 |
| *P. gingivalis* | 0 |
| *S. mitis* | 0.2 |

**Supplementary methods 4. Assay IDs used in the quantitative TaqMan PCR assays**

| **Assay ID** | **Gene Symbol(s)** | **Gene Name(s)** | **Amplicon Length** |
| --- | --- | --- | --- |
| Hs99999901_s1 | 18s rRNA | 18S | 187 |
| Hs00201226_m1 | CASC3 | cancer susceptibility candidate 3 | 67 |
| Hs00174128_m1 | TNF | tumor necrosis factor | 80 |
| Hs00174131_m1 | IL6 | interleukin 6 | 95 |
| Hs00354519_m1 | CD36 | CD36 molecule | 83 |
| Hs00968305_m1 | MMP3 | matrix metallopeptidase 3 | 126 |
| Hs00765553_m1 | CCND1 | cyclin D1 | 57 |
| Hs02387400_g1 | NANOG | Nanog homeobox | 109 |

**Supplementary methods 5. Intracellular and extracellular bacteria Staining and Microscopy**

Staining of intracellular versus extracellular bacteria has been adapted as previously described (1). CAL27, SCC25, and SCC4 cells were seeded onto 24-well tissue culture plates at 10x10^6, 8x10^6, 8x10^6 cells per well, respectively. Bacteria (*N. flavescens* and *H. parainfluenzae*) were washed twice with sterile PBS and labeled using FAM-CFSE (ThermoFisher C1311) at 10µg/ml for 30’ in a tube rotator at room temperature. Bacteria were then incubated with 1mg/ml of Sulfo-NHS-LC-biotin (ThermoFisher 21335) for 30’ at room temperature. Bacteria were then washed three times with PBS before infection of CAL27, SCC25, and SCC4 at MOI of 100 for 24h. Infected cells were washed three times with PBS and blocked with PBS 10% FBS for 5’. Extracellular bacteria were stained for 45’ at room temperature with Streptavidin-Alexa647 (ThermoFisher S21374) at 1:200 in blocking buffer. Cells were washed three times with PBS. DNA was stained with NucBlue Live (ThermoFisher R37605) for 5’. Cells were washed three times with PBS before imaged using EVOS2 FL Auto microscope (ThermoFisher).

**Supplementary methods 6. Antibiotic protection assays**

CAL27, SCC25, and SCC4 cells were seeded onto 24-well tissue culture plates at 10x10^6, 8x10^6, 8x10^6 cells per well, respectively. Actively growing *N. flavescens* and *H. parainfluenzae* cells were pelleted by centrifugation at 5000 rpm for 5 minutes, washed three times in phosphate-buffered saline (PBS), and suspended in DMEM media supplemented with 10% fetal bovine serum (FBS) and 2.5 mM L-glutamine and were added at MOI 100 to the OSCC cells seeded 24 hours before. Penicillin/streptomycin was included in the culture medium at sub-minimum inhibitory concentration (sub-MIC) mentioned in the Supplementary method 3 A. 24 hours after addition of bacteria, culture media was replaced with media containing 100 IU/ml penicillin/streptomycin mix and was incubated for 4 hours to kill all the extracellular bacteria. After 4 hours the media was replaced and the wells were washed with PBS, 10 µl of PBS from washed wells were spot plated on the BHKI culture plate to check the viability of extracellular bacteria, if any. After washing a lysis solution containing 0.3% Tween 20 in PBS containing 25% of 0.25% Trypin-EDTA solution was added to the wells and were incubated for 20 minutes at 37% in a CO_2_ incubator. After 20 minutes’ plate was removed from incubator and the cell-lysis mixture was mixed well by pipetting up and down atleast 10 times. The lysate from the wells were transferred to 1.5 ml eppendorf tubes, centrifuged at 12000 rpm for 5 minutes, supernatant was discarded and the pellet was suspended in PBS. Serial dilution of the suspended cells was spot plated on BHKI agar plates and was incubated for 24 hours at 37°C and the colonies were counted to obtain the CFU for each bacteria from the OSCC cell lines.

**Supplementary methods 7. Quantification of IL6 and TNF α**

Culture supernatants from the OSCC cells exposed to MOI 100 of *H. parainfluenzae*, *N. flavescens*, *S. mitis* or *P. gingivalis* were collected after 24 hours and immediately stored at -80°C till the protein analysis. IL-6 and TNF-α biomarker analysis was performed by Eve Technologies Corp. (Calgary, Alberta) using the Human High Sensitivity 14-Plex Discovery Assay® (MilliporeSigma, Burlington, Massachusetts, USA) on a Luminex™ 200 system (Luminex, Austin, TX, USA).

**Reference**

1. F. Agerer, S. Waeckerle, C. R. Hauck, Microscopic quantification of bacterial invasion by a novel antibody-independent staining method. *J Microbiol Methods* **59**, 23-32 (2004).
