# Supplementary material for: Screening of Health-Associated Oral Bacteria for Anticancer Properties in vitro
Source: Front Cell Infect Microbiol. 2020 Oct 6;10:575656. doi: 10.3389/fcimb.2020.575656 (PMC7573156; doi:10.3389/fcimb.2020.575656)
Supplement: Supplementary file 3 [file Data_Sheet_2.docx]

**Supplementary Table 1.** Luminescence values in the Caspase-Glo® 3/7 Assay (Promega, USA) after 5 hours of co-culture of OSCC cells with *S. mitis*,

|  | Control | MOI 10 | MOI 50 |
| --- | --- | --- | --- |
| CAL27 | 3881.3 | 5657.7* | 3526.0 |
| SCC 25 | 8018.7 | 8128.0 | 8890.0* |
| SCC4 | 8046.7 | 9007.3* | 9930.0* |

All the treatments were performed in triplicates in a 96-well plate. Luminescence values are directly proportional to caspase activity indicative of apoptosis. * Differences are significant compared to the control as measured by pairwise t test (p<0.05)

|  | Control | MOI 10 | MOI 50 |
| --- | --- | --- | --- |
| CAL27 | 608.3 | 4944.3* | 5519.3* |
| SCC25 | 1328.0 | 1458.5* | 4219.3* |
| SCC4 | 858.0 | 6773.0* | 7252.0* |

**Supplementary Table 2.** Fluorescence values in RealTime-Glo™ Annexin V Apoptosis and Necrosis assay kit (Promega, USA) after 20 hours of co-culture of OSCC cells with *S. mitis*.

All the treatments were performed in triplicates in a 96-well plate. Fluorescence values are directly proportional to loss of cell membrane indicative of necrosis (late apoptosis). * Differences are significant compared to the control as measured by pairwise t test (p<0.05)

**Supplementary Table 3**. Experiment demonstrating intracellular infection of SCC4 by *N. flavescens*

| Condition | OD_600_ | | |
| --- | --- | --- | --- |
|  | **24 h** | **48h** | **72 h** |
| Bacteria inoculated in culture medium with no antibiotics - no cells seeded | 0.368 | 0.392 | 0.369 |
| Bacteria inoculated in culture medium with sub MIC antibiotics - no cells seeded | 0.056 | 0.054 | 0.057 |
| No bacteria inoculated in culture medium with sub MIC antibiotics – cells seeded | 0.053 | 0.055 | 0.055 |
| Bacteria inoculated in culture medium with sub MIC antibiotics – cells seeded | 0.234 | 0.273 | 0.331 |

All the treatments were performed in triplicates in a 48-well plate and the treatments were incubated at in 5% CO_2_ at 37 ̊C for 72 hours and OD600 was measured after 24, 48 and 72 hours.

**Supplementary Table 4**. Experiment demonstrating intracellular infection of CAL27 by *N. flavescens*

| Condition | OD_600_ | | |
| --- | --- | --- | --- |
|  | **24 h** | **48h** | **72 h** |
| Bacteria inoculated in culture medium with no antibiotics - no cells seeded | 0.212 | 0.339 | 0.305 |
| Bacteria inoculated in culture medium with sub MIC antibiotics - no cells seeded | 0.065 | 0.078 | 0.064 |
| No bacteria inoculated in culture medium with sub MIC antibiotics – cells seeded | 0.054 | 0.051 | 0.061 |
| Bacteria inoculated in culture medium with sub MIC antibiotics – cells seeded | 0.164 | 0.215 | 0.175 |

All the treatments were performed in triplicates in a 48-well plate and the treatments were incubated at in 5% CO_2_ at 37 ̊C for 72 hours and OD600 was measured after 24, 48 and 72 hours.

**Supplementary table 5.** Average number of CFUs recovered from OSCC cell lines after 24 hours of co-culture with *H. parainfluenzae*

|  | # average CFUs |
| --- | --- |
| CAL27 | 1435.556 |
| SCC25 | 377.7778 |
| SCC4 | 13222.22 |

No-bacteria controls and post-antibiotic exposure PBS wash controls (extracellular bacteria) did not show any growth. All the treatments were performed in triplicates in a 24-well plate and from each replicate, spot plating was performed in triplicates again to count CFUs.

**Supplementary table 6.** Average number of CFUs recovered from OSCC cell lines after 24 hours of co-culture with *N. flavescens*

|  | # average CFUs |
| --- | --- |
| CAL27 | 1386.667 |
| SCC25 | 357.7778 |
| SCC4 | 15400 |

No-bacteria controls and post-antibiotic exposure PBS wash controls (extracellular bacteria) did not show any growth. All the treatments were performed in triplicates in a 24-well plate and from each replicate, spot plating was performed in triplicates again to count CFUs.
